# Piscine Orthoreovirus-1 Isolates Differ in Their Ability to Induce Heart and Skeletal Muscle Inflammation in Atlantic Salmon (*Salmo salar*)

**DOI:** 10.3390/pathogens9121050

**Published:** 2020-12-14

**Authors:** Øystein Wessel, Elisabeth F. Hansen, Maria K. Dahle, Marta Alarcon, Nina A. Vatne, Ingvild B. Nyman, Karen B. Soleim, Kannimuthu Dhamotharan, Gerrit Timmerhaus, Turhan Markussen, Morten Lund, Håvard Aanes, Magnus Devold, Makoto Inami, Marie Løvoll, Espen Rimstad

**Affiliations:** 1 Faculty of Veterinary Medicine, Norwegian University of Life Sciences, 0454 Oslo, Norway; elisabeth.hansen@nmbu.no (E.F.H.); nina.askim.vatne@nmbu.no (N.A.V.); ingvild.nyman@nmbu.no (I.B.N.); dhamubfsc@gmail.com (K.D.); turhan.markussen@nmbu.no (T.M.); espen.rimstad@nmbu.no (E.R.); 2 Norwegian Veterinary Institute, 0454 Oslo, Norway; maria.dahle@vetinst.no (M.K.D.); karen.bakken-soleim@vetinst.no (K.B.S.); 3 FishVetGroup, 0275 Oslo, Norway; marta.alarcon@fishvetgroup.com; 4 Nofima, 1430 Ås, Norway; gerrit.timmerhaus@nofima.no; 5 PatoGen, 6002 Ålesund, Norway; morten@patogen.no (M.L.); havard@patogen.no (H.A.); magnus@patogen.no (M.D.); 6 VESO Vikan, 7810 Namsos, Norway; makoto.inami@veso.no (M.I.); marie.lovoll@veso.no (M.L.)

**Keywords:** PRV-1, heart and skeletal muscle inflammation, virulence, Atlantic salmon

## Abstract

Piscine orthoreovirus 1 (PRV-1) is the causative agent of heart and skeletal muscle inflammation (HSMI) in farmed Atlantic salmon (*Salmo salar*). The virus is widespread in Atlantic salmon and was present in Norway long before the first description of HSMI in 1999. Furthermore, in Canada the virus is prevalent in farmed Atlantic salmon but HSMI is not and Canadian isolates have failed to reproduce HSMI experimentally. This has led to the hypothesis that there are virulence differences between PRV-1 isolates. In this study we performed a dose standardized challenge trial, comparing six PRV-1 isolates, including two Norwegian field isolates from 2018, three historical Norwegian isolates predating the first report of HSMI and one Canadian isolate. The Norwegian 2018 isolates induced lower viral protein load in blood cells but higher plasma viremia. Following peak replication in blood, the two Norwegian 2018 isolates induced histopathological lesions in the heart consistent with HSMI, whereas all three historical Norwegian and the Canadian isolates induced only mild cardiac lesions. This is the first demonstration of virulence differences between PRV-1 isolates and the phenotypic differences are linked to viral proteins encoded by segment S1, M2, L1, L2 and S4.

## 1. Introduction

Piscine orthoreovirus 1 (PRV-1) is the causative agent of heart and skeletal muscle inflammation (HSMI), an important disease in farmed Atlantic salmon (*Salmo salar*) [1,2,3]. However, all PRV-1 infected fish do not develop HSMI. This indicates that unknown viral, host or environmental factors are important for disease development.

In 1999, the first outbreak of HSMI was described in farmed Atlantic salmon in Norway [1]. Subsequently, the disease spread along the coast and is now causing numerous outbreaks each year in Norway [4]. The disease is characterized mainly by inflammation in the heart and skeletal muscle, with outbreaks of high morbidity and mortality up to 20% [2]. PRV-1 was identified in diseased fish [3] and later shown to be the causative agent of HSMI [2]. Interestingly, the virus has been revived from Norwegian archival samples dating back to 1988 [5], 11 years prior to the appearance of the disease. This has led to a hypothesis that virus evolution has increased the virulence of PRV-1, which in turn has contributed to the emergence of HSMI in Norway [5].

PRV-1 is almost ubiquitous in countries with large scale farming of Atlantic salmon but HSMI does not appear to be. Westcoast Canada is a notable example; the virus is readily detected in farms but only a few cases of HSMI have been reported [6]. Similarly, the Faroe Islands have reported the virus but not the disease [5]. This contrasts the high prevalence of HSMI observed in Norway [4], as well as observations of HSMI in Scotland and Chile [7,8].

Experimental trials performed with Norwegian and Canadian PRV-1 isolates have given different results. HSMI has been reproduced with Norwegian isolates in a number of experimental trials [2,9,10,11], whereas similar experiments with Canadian isolates have not [12,13]. All trials describe an early acute phase characterized by high viral load in blood [2,12,13], when PRV-1 replicates in its major target cell erythrocytes [14]. The major difference is the subsequent development of heart lesions observed with Norwegian isolates [2,9], which is almost absent with Canadian isolates [12,13]. Furthermore, challenge experiments with Canadian isolates have reported no virus in plasma and only minor antiviral immune responses during replication in blood [12,13]. Similar experiments with Norwegian isolates have shown high plasma viremia and a robust innate immune response in blood cells [2,15]. These observations have led to a hypothesis that there are virulence differences between Norwegian and Canadian PRV-1 isolates. However, experimental testing of the hypothesis requires that the isolates are compared in a trial where host and environmental factors as well as viral inoculum are all standardized.

Differences in virulence are reflected in the nucleotide sequence of the viral genome. PRV-1 belongs to the genus *Orthoreovirus* in the family Reoviridae [16,17] and has a ten-segmented double-stranded RNA (dsRNA) genome packed in a double protein capsid [18]. The three large (L1–3) three medium (M1–3) and four small (S1–4) segments each encodes one protein named with its corresponding Greek letter λ, μ and σ, except segment S1, which is biscistronic [16]. Through point mutations, reassortment and recombination these viruses will naturally explore the available sequence space and natural selection will continuously optimize their fitness to changing intra- and extracellular conditions [19]. Phenotypical properties, such as induction of HSMI, can be linked to single or multiple mutations or reassortment involving one or several segments. PRV-1 sequences that are available in accessible depositaries mostly include only the S1 sequence and without a description of the disease history of the fish population from where the virus isolate originated, which together limits the utility of these nucleotide sequences in virulence investigations. The number of whole genome PRV-1 sequences are increasing and in silico studies have identified segment S1 and M2 as important for the development of HSMI [5]. Furthermore, comparison of Norwegian and Pacific Canadian isolates used in challenge trials have shown that most sequence variation is found in segment S1 [12]. 

The aim of this study was to determine virulence differences in Atlantic salmon between PRV-1 isolates. Due to PRV-1′s resistance to be propagated in cell cultures, recent methodologic advancement for virus purification was utilized to standardize the challenge dose in a trial comparing two Norwegian field isolates from 2018, three historical Norwegian isolates predating discovery of HSMI and one Canadian isolate. The results demonstrated virulence differences between PRV-1 isolates regarding their ability to induce HSMI.

## 2. Results

Six PRV-1 isolates were compared in a standardized challenge trial: two Norwegian field isolates collected from farmed Atlantic salmon in 2018 (NOR-2018/SF, NOR-2018/NL), three historical Norwegian isolates predating discovery of HSMI in Norway (NOR-1997, NOR-1996, NOR-1988) and one Canadian isolate from British Colombia (CAN 16-005ND).

### 2.1. Viral Load in Blood Cells Differ between Isolates

**Figure 1:**
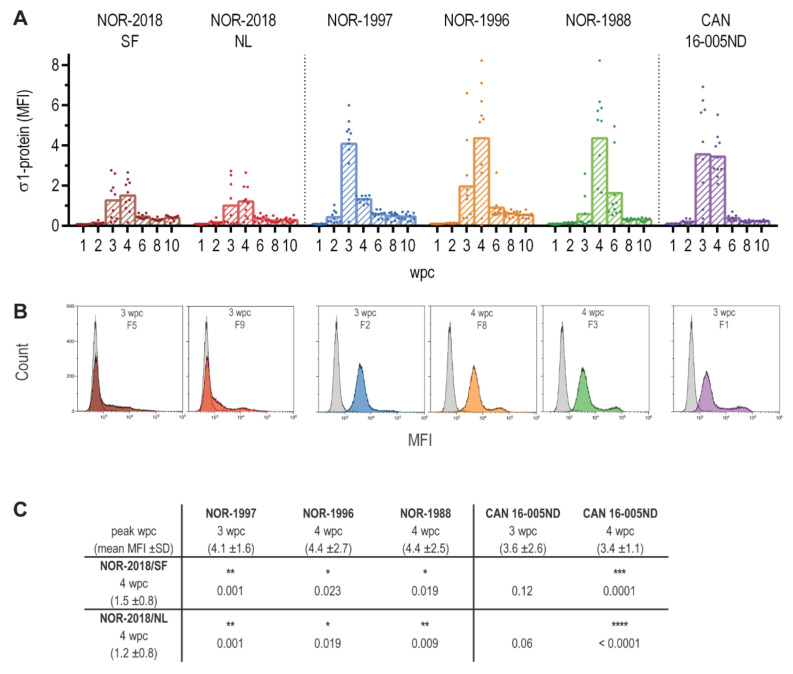
PRV σ1-protein in blood cells. (**A**) Amount of σ1-protein in blood cells measured by flow cytometry from 1 to 10 weeks post challenge (wpc), shown as mean fluorescence intensity (MFI) for individual fish and group mean for NOR-2018/SF (dark red), NOR-2018/NL (red), NOR-1997 (blue), NOR-1996 (orange), NOR-1988 (green) and CAN 16-005ND (purple). (**B**) Histogram from one individual fish at peak of infection from each group, MFI for σ1-protein on X-axis and cell count on y-axis, counting 30,000 cells per sample. Color coding as above, overlaid PRV negative control fish (grey). (**C**) Statistical analysis comparing mean MFI at peak of infection (mean MFI ± SD shown) for the NOR-2018/SF and NOR-2018/NL, to that of NOR-1997, NOR-1996, NOR-1988 and CAN 16-005ND using Mann-Whitney test, results shown as *p*-values (* *p* < 0.05, ** *p* < 0.01, *** *p* < 0.001, **** *p* < 0.0001).

In the first phase of infection, PRV-1 targets erythrocytes. Replication of the different virus isolates in erythrocytes was assessed by measuring the viral load in blood cells by flow cytometry detecting the σ1-protein. All groups reached the peak of infection at 3–4 weeks post challenge (wpc) but the viral load was significantly lower in the two NOR-2018 isolates (NOR-2018/SF, NOR-2018/NL), compared to the historical Norwegian (NOR-1997, NOR-1996, NOR-1988) and the Canadian isolate (CAN 16-005ND) (Figure 1A,C). Furthermore, at peak of infection viral protein was only detected in a subpopulation of the blood cells in the Norwegian 2018 isolates, whereas all blood cells were positive in the historical Norwegian and Canadian isolates (Figure 1B). Post peak of infection, the load of viral protein dropped substantially in all groups (Figure 1A).

The level of viral RNA in blood cells was also measured by reverse transcription-quantitative polymerase chain reaction (RT-qPCR) targeting the S1 segment. The peak load of viral RNA coincided in time to that of viral protein but the RT-qPCR results did not reveal significant differences between the virus isolates as observed in load of viral protein (Supplementary Figure S1). Furthermore, post peak infection the levels of viral RNA remained relatively high in contrast to the level of viral protein which dropped rapidly.

### 2.2. Transient Reduction in Hemoglobin Concentration

**Figure 2:**
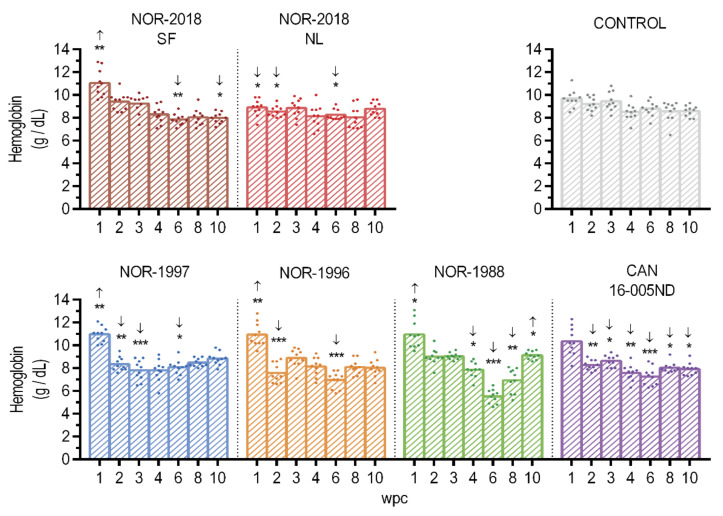
Hemoglobin. Concentration of hemoglobin presented as individual and mean value from 1 to 10 weeks post challenge (wpc). Statistical analysis comparing each isolate (color coded) to the control (grey) at each time point using Mann-Whitney test. Significant differences presented as * *p* < 0.05, ** *p* < 0.01, *** *p* < 0.001, including arrow showing if it was higher (up) or lower (down) compared to the control group.

To determine if there was variation in the isolates’ ability to cause anemia the hemoglobin concentration in blood was measured. The hemoglobin concentration was reduced in all groups at or just after peak infection in blood compared to the control group (Figure 2). In the NOR-1997, the lowest concentration was observed at 3 wpc, whereas at6 wpc for NOR-1996, NOR-1988 and CAN 16-005ND. A drop was also observed at 6 wpc for the NOR-2018/SF and NOR-2018/NL isolates compared to the control group, but the concentration did not seem to be severely reduced. At the end of the study the hemoglobin concentration started to recover in all groups. It should be noted that the hemoglobin level also varied prior to onset of replication in blood (1–2 wpc) (Figure 2).

### 2.3. High-Level Plasma Viremia after Infection with Norwegian 2018 Isolates

**Figure 3:**
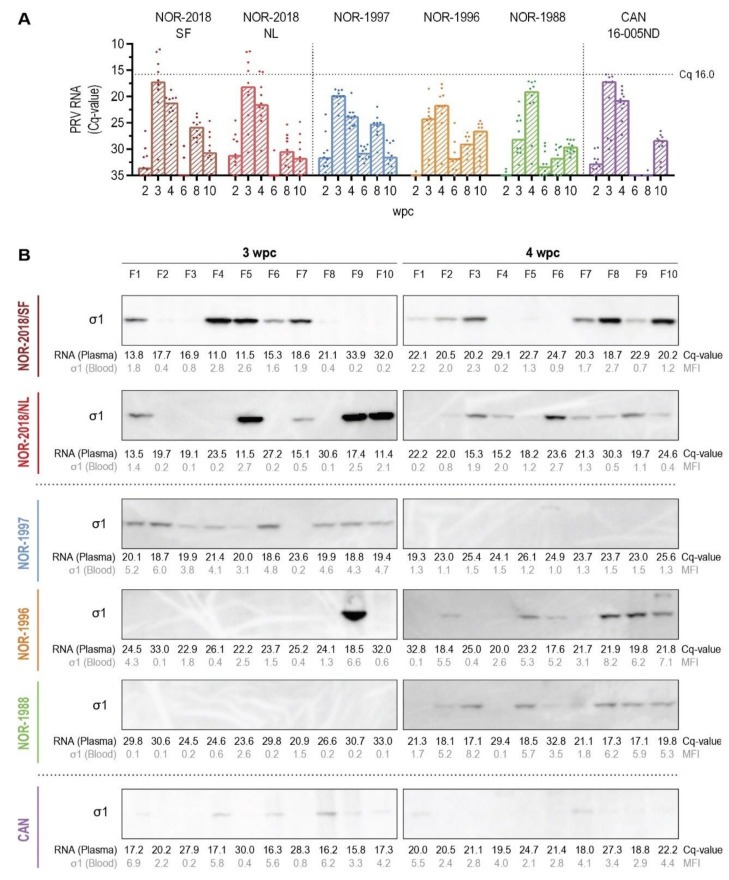
Plasma viremia. (**A**) PRV RNA in plasma measured by reverse transcription-quantitative polymerase chain reaction (RT-qPCR), shown as individual and median Cq-values from 2 to 10 weeks post challenge (wpc). Horizontal dotted line marks Cq 16.0. (**B**) PRV σ1-protein detected in plasma by Western Blotting at 3 and 4 wpc from fish F1–10 for each group. Listed below the blots are the viral RNA loads in plasma (Cq-value) and σ1-protein loads in blood cells (MFI) from each individual fish.

To compare the amount of virus released following replication in erythrocytes, the level of virus in plasma was measured by detection of viral RNA (RT-qPCR) and viral protein (western blotting). High loads of viral RNA in plasma were found for the Norwegian 2018 isolates at 3 wpc, with median Cq 17.3 (IQR 13.2-23.8) and 18.3 (IQR 13.0-24.4) for NOR-2018/SF and NOR-2018/NL, respectively (Figure 3A). At this time point, four out of ten fish presented with Cq-value below 16.0, with maximum Cq of 11.0 (NOR-2018/SF) and 11.4 (NOR-2018/NL). At 4 wpc, the viral load was still relatively high but with a higher median Cq-value and without the extreme loads observed at 3 wpc (Figure 3A).

On the other hand, viral RNA in plasma of the historical Norwegian and the Canadian isolates also peaked at 3–4 wpc but with the notable absence of individuals with Cq below 16, except one fish in the Canadian group at Cq 15.8. The NOR-1997 and Canadian isolate peaked at 3 wpc with median Cq of 19.9 (IQR 18.8-20.4) and 17.3 (IQR 16.3-28.0) respectively, whereas NOR-1996 and NOR-1988 peaked at 4 wpc with median Cq of 21.8 (IQR 19.5-23.7) and Cq 19.2 (IQR 17.3-23.3) respectively. In general, the RNA detection in plasma was characterized by rather large variation within all groups. The RNA levels in plasma in both Norwegian 2018 isolates was significantly different at 3 wpc to NOR-1996 and NOR-1988 (*p* ≤ 0.05) and to NOR-1997 at 4 wpc (*p* ≤ 0.05) but not to the Canadian isolate at any of the time points (Supplementary Table S1). Post peak of infection (6 wpc) the level of viral RNA dropped substantially in all groups, with little or no virus detection and thereafter increased moderately at 8 or 10 wpc in all groups (Figure 3A).

To validate the viral RNA detection in plasma at peak of infection, the samples were analyzed for PRV σ1-protein by western blotting. In both Norwegian 2018 isolates, high loads of σ1-protein were observed in plasma (Figure 3B). At 3 wpc the viral protein was detected in five out of ten fish in both groups, observed as bands with strong or moderate intensity (Figure 3B). At 4 wpc, seven out of ten fish were positive, with moderate or faint band intensity. In comparison, the historical Norwegian and the Canadian isolate generally presented with lower loads of σ1-protein in plasma (Figure 3B). In NOR-1997 infected fish, only faint bands were detected at 3 wpc (nine out of ten) and at 4 wpc no bands were observed. In NOR-1996 and NOR-1988 infected fish no bands were observed at 3 wpc, except one strong band in NOR-1996 and at 4 wpc faint bands were observed in both groups (six out of ten). Similarly, in fish infected with the Canadian isolate only faint bands were observed at 3 wpc (six out of ten) and at 4 wpc (two out of ten).

In general, the detection of viral protein correlated with that of viral RNA in plasma (Figure 3B). The plasma samples with highest RNA loads, mainly observed in Norwegian 2018 isolates at 3 wpc, also showed strong bands in western blot. In samples with lower RNA levels (Cq-values 20–25), faint bands or negative blots were observed and no bands were observed in plasma samples with Cq values above 25. The presence of viral protein in plasma was verified by western blotting for the structural µ1 and λ1 proteins in the NOR-2018/SF and Canadian groups. In brief, higher loads of µ1 and λ1 were observed in NOR-2018/SF, that is, a similar pattern as the σ1-protein but with fainter bands (Supplementary Figure S2).

Interestingly, although the load of structural proteins in plasma were consistently higher in the NOR-2018/SF group compared to the Canadian group, the load of the non-structural protein σNS was relatively similar. In both the NOR-2018/SF group and Canadian group, σNS were detected with strong band intensity in a number of samples both at 3 and 4 wpc. This coincided with high loads of structural proteins in the NOR-2018/SF group, whereas no or only faint bands were observed for structural proteins in the Canadian group (Supplementary Figure S2).

### 2.4. Cardiac Scores are Significantly Higher following Infection with the Norweigan 2018 Isolates

**Figure 4:**
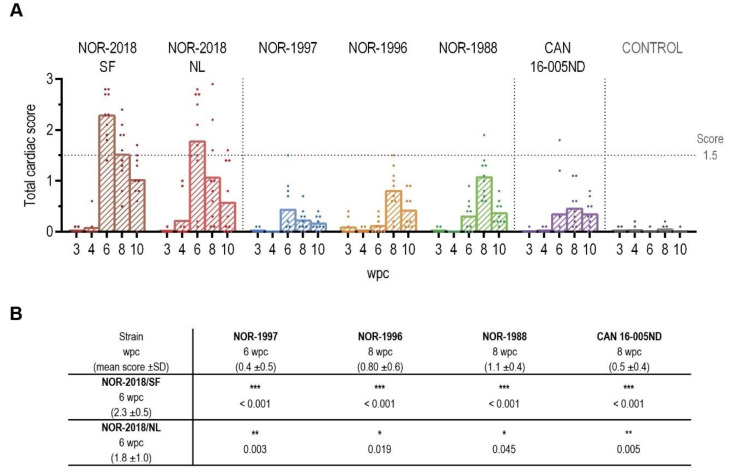
Heart histopathology scoring. (**A**) Total cardiac score shown as individual score and group mean from 3 to 10 weeks post challenge (wpc) for the six different PRV groups (color coded) and the control group (grey) (*n* = 10). Horizontal dotted line marks score 1.5. (**B**) Statistical analysis comparing mean total cardiac score at peak of infection of NOR-2018/SF and NOR-2018/NL to that of NOR-1997 NOR-1996, NOR-1988 and CAN 16-005ND using Mann-Whitney test, results shown as *p*-values (* *p* < 0.05, ** *p* < 0.01, *** *p* < 0.001).

**Figure 5:**
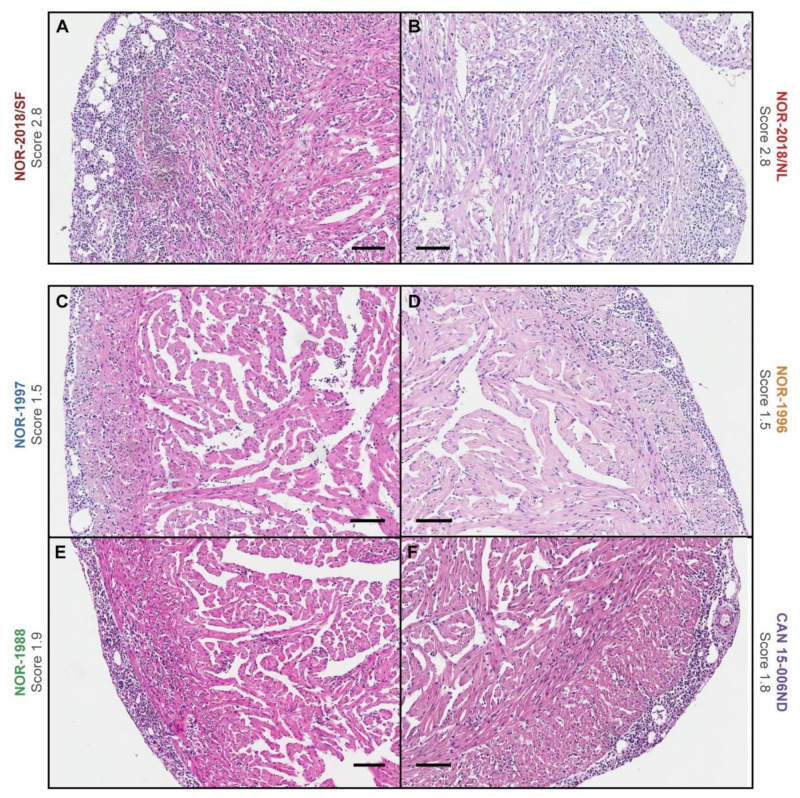
Heart histopathology. Histopathology image of the heart with the highest cardiac score observed in each group, including (**A**) NOR-2018/SF, (**B**) NOR-218/NL, (**C**) NOR-1997, (**D**) NOR-1996, (**E**) NOR-1988 and (**F**) CAN 16-005ND. Total cardiac score shown next to group title. Scale bar 100 μm.

To compare the ability of each isolate to induce HSMI, the hearts were scored for cardiac lesions. Infection with the two Norwegian 2018 isolates gave severe histopathological lesions in the heart at 6 wpc. The NOR-2018/SF group had a mean total cardiac score of 2.3 ± 0.5 (scale from 0–3) and all ten fish demonstrated cardiac lesions in the range 1.4 to 2.8 (Figure 4A). The NOR-2018/NL group had a mean total cardiac score of 1.8 ± 1.0 where eight out of ten fish scored 1.4 to 2.8 and two fish had little or no lesions (Figure 4A). The lesions were characterized by severe lymphocytic inflammation in the epicardium, compactum and spongium and moderate inflammation in the atrium, consistent with the lesions observed during HSMI (Figure 5). Scoring of each compartment throughout the study is shown in Supplementary Figure S3. After the peak of lesions there was a reduction in total cardiac score in both NOR-2018 groups (Figure 4A).

In the historical Norwegian and Canadian isolate, significantly lower cardiac scores were observed compared to the Norwegian 2018 isolates (Figure 4A,B). In the NOR-1997 group, peak lesions were observed at 6 wpc with mean total cardiac lesions of 0.4 ± 0.5, whereas in the NOR-1996 and NOR-1988 groups, little or no lesions were observed at 6 wpc and peak lesions were observed at 8 wpc, with total mean cardiac score of 0.8 ± 0.6 and 1.1 ± 0.4 respectively. In the Canadian group, mean total cardiac score were increased at 6 wpc and peaked at 8 wpc with a score of 0.5 ± 0.4 (Figure 4A). In general, the histopathological lesions in the historical Norwegian and Canadian isolate were similar to that observed for the Norwegian 2018 isolates but less severe. However, they were significantly higher than the control group in which little or no cardiac lesions were observed (Figure 4A). In NOR-1997, NOR-1996, NOR-1988 and the CAN 16-005ND, a few individual fish with lesions of 1.5 or higher were observed, thus within the range of observed at peak of infection with the NOR-2018/SF and NOR-2018/NL (Figure 5). For comparison, histopathological images of all fish at peak of infection are presented in Supplementary Figures S4–S9.

### 2.5. Higher Load of PRV RNA in Cardiomyocytes following Infection with Norwegian 2018 Isolates

**Figure 6:**
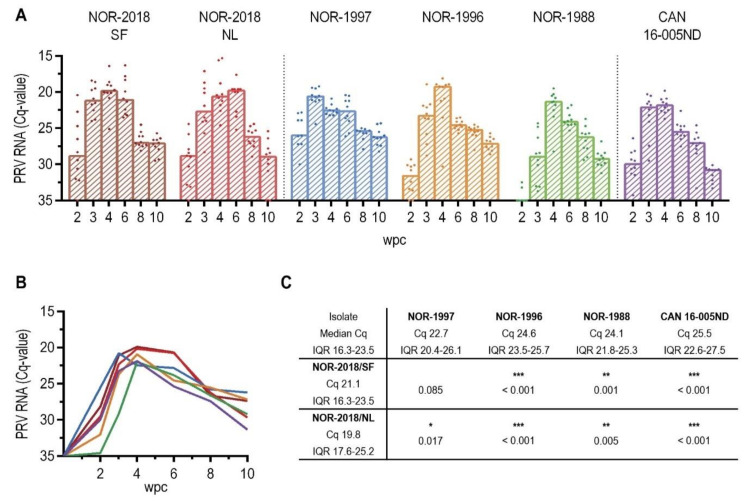
PRV RNA in heart by RT-qPCR. (**A**) PRV RNA in heart measured by RT-qPCR, shown as individual and median Cq-values from 2 to 10 weeks post challenge (wpc), (**B**) also shown as line graph with median Cq-values. (**C**) Statistical analysis comparing median Cq-values at peak RNA loads in heart for NOR-2018/SF and NOR-2018/NL to that of NOR-1997 NOR-1996, NOR-1988 and CAN 16-005ND using Mann-Whitney test, results shown as *p*-values (* *p* < 0.05, ** *p* < 0.01, *** *p* < 0.001).

We hypothesized that a higher cardiac score was associated with higher viral loads in the heart. Therefore, we measured the level of PRV RNA in the heart by RT-qPCR. In the early phases relatively similar kinetic was observed in all groups, reaching peak loads at 4 wpc, except NOR-1997 which peaked at 3 wpc (Figure 6A). However, at 6 wpc, higher loads of viral RNA were observed for the Norwegian 2018 isolates, coinciding with the development of severe cardiac lesions (Figure 6A,B). The load was significantly higher at peak of infection compared to that of the historical Norwegian and Canadian isolates, except for NOR-2018/SF compared to NOR-1997 (Figure 6C).

**Figure 7:**
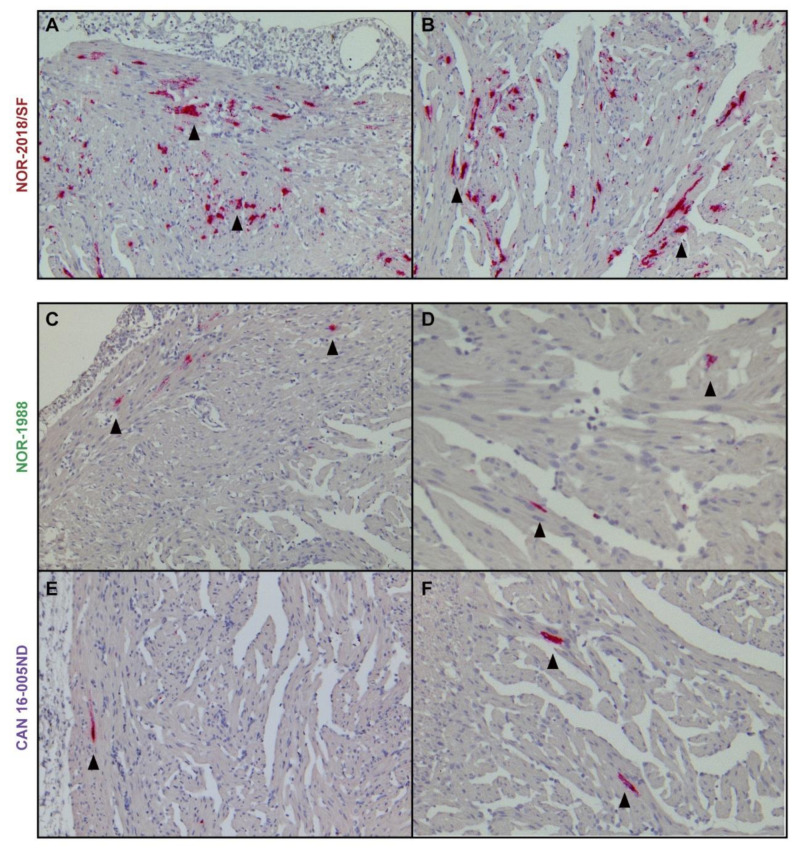
PRV detection in cardiomyocytes. Detection of PRV RNA by in-situ hybridization in cardiomyocytes after infection with NOR-2018/SF (**A**,**B**), NOR-1988 (**C**,**D**) and CAN 16-005ND (**E**,**F**). Positive staining observed as red staining and marked by arrowheads. Images of compactum and spongiosum layer presented in left (**A**,**C**,**E**) and spongiosum layer on the right side (**B**,**D**,**F**).

To further evaluate the viral load in cardiomyocytes, in-situ hybridization for PRV RNA was performed from 4–8 wpc, testing one isolate which induced severe cardiac lesions (NOR-2018/SF) and two isolates which induced mild cardiac lesions (NOR-1988 and CAN 16-005ND). In the NOR-2018/SF isolate, PRV was detected in cardiomyocytes at 4 wpc as punctuated staining and at 6 wpc numerous cardiomyocytes with strong signal intensity was observed in both the compact and spongy layer (Figure 7A,B). At 8 wpc only scattered single positive cardiomyocytes were detected. In general, fewer positive cardiomyocytes were observed after infection with NOR-1988 (Figure 7C,D) and the Canadian isolate (Figure 7E,F). At 4 wpc, the cardiomyocytes were mainly negative and at 6 wpc and 8 wpc PRV positive cardiomyocytes were observed but predominantly as small focal areas or scattered single cells.

The in-situ staining also demonstrated positive blood cells within the heart. This was mainly observed at 4 wpc, with numerous blood cells which often appeared to be attached to the endothelium. This was observed after infection with all three PRV-1 isolates tested, including NOR-2018/SF, NOR-1988 and CAN 16-005ND (Supplementary Figure S10). At 6 and 8 wpc the number of positive blood cells in the heart was substantially lower in all groups.

### 2.6. Strong Antiviral Response in Blood Cells Induced by All Isolates

**Figure 8:**
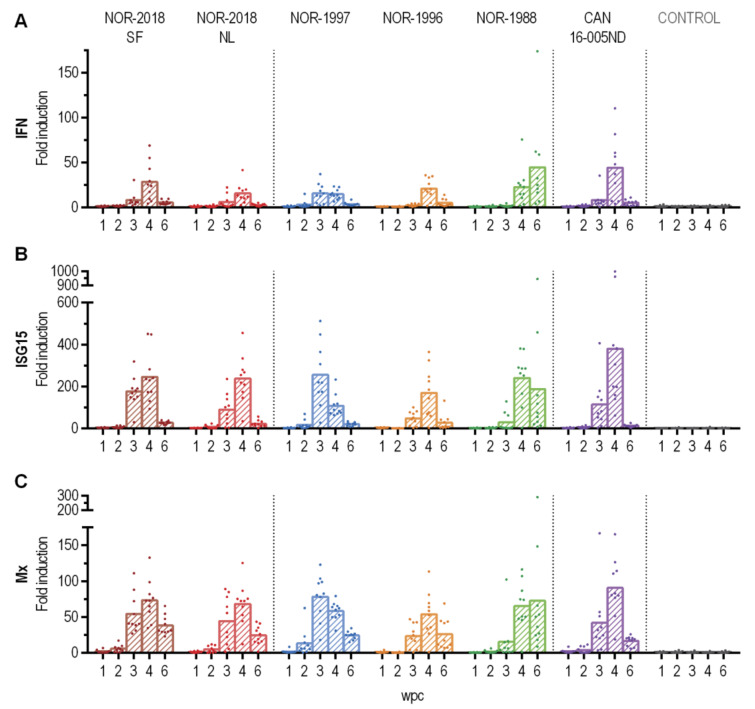
Expression of innate antiviral genes in blood cells. Relative expression of (**A**) type I interferon (IFNab), (**B**) Interferon stimulated gene 15 (ISG15) and (**C**) myxovirus resistance GTPase (Mx1) for six PRV-1 isolates (color coded) and non-infected controls (grey) from 1–6 weeks post challenge (*n* = 10). Target Cq-values are normalized against the expression level of elongation factor (EF) 1α and fold induction is shown relative to the mean expression level in control fish at each sampling time point.

To compare the innate antiviral response in blood cells after infection with the different PRV-1 isolates, the expression of a selection of innate immune genes were assessed by RT-qPCR. No substantial differences in expression levels were observed between the two Norwegian 2018 isolates, historical Norwegian (NOR-1997, NOR-1996, NOR-1988) or the Canadian isolate. In general, transcription of type I interferon (primers targeting IFNab), interferon-stimulated gene 15 (ISG15) and myxovirus resistance GTPase (Mx1) was up-regulated at 3–4 wpc in all groups and reduced at 6 wpc (Figure 8). The three innate antiviral genes followed a similar expression pattern and the up-regulation coincided with the peak of infection in each group. 

### 2.7. Virulence Differences Linked to Segment L1, L2, M2, S1 and S4

**Figure 9:**
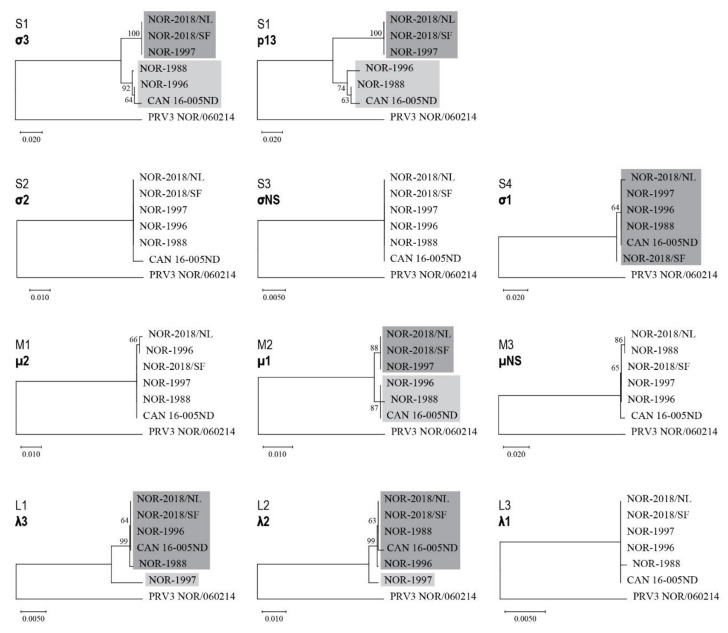
Phylogenetic trees constructed from the eleven known amino acid sequences encoded by PRV-1 using neighbor joining (NJ), for all six isolates studied (NOR-2018/NL, NOR-2018/SF, NOR-1997, NOR-1996, NOR-1988 and CAN 16-005ND). Protein sequences potentially linked to virulence are colored grey; dark grey to mark variants associated with higher virulence, light grey for lower virulence. Bootstrap values were calculated from 1000 replicates and values above 70 can be considered significant [20]. PRV-3 strain NOR/060214 (MG253807-MG253816) was selected as the outgroup.

Phylogenetic analysis of all six PRV-1 isolates were performed for each genome segment (Supplementary Figure S11) and for the encoded amino acid sequences (Figure 9) and combined with analysis aiming to identify viral sequences linked to the phenotypical differences observed in the challenge trial.

For the S1-encoded σ3 and p13 proteins and the M2-encoded μ1, the six isolates form two separate phylogenetic groups with high bootstrap support (Figure 9). One group consisted of the two Norwegian 2018 isolates and the historical NOR-1997, all carrying identical amino acid sequences (Supplementary Table S2). The other group contained the historical Norwegian isolates NOR-1988 and NOR-1996 and the Canadian isolate (CAN 16-005ND) (Figure 9). A similar grouping of the six isolates was observed in the phylogenetic trees constructed from the S1 and M2 genomic sequences, although with somewhat lower bootstrap support for one of the groups (Supplementary Figure S11). The two phylogenetic groups for the σ3, p13 and u1 proteins (and gene segments) is reflected in the pattern of shared amino acid differences between the two groups (Supplementary Table S2).

For the λ3 and λ2 proteins, encoded by segment L1 and L2 respectively, the NOR-1997 isolate stands out by not grouping together with the other five isolates, which form a monophyletic group with high bootstrap support for both proteins (Figure 9). The same pattern was observed in the phylogenetic trees constructed from the L1 and L2 genomic sequences, also with high bootstrap support (Supplementary Figure S11). A comparison of the amino acid sequences revealed that 13 (λ3) and 10 (λ2) differences are unique to NOR-1997 (Supplementary Table S2). 

Analysis of the S4-encoded σ1 sequences showed that they were identical for the three historical Norwegian isolates and the Canadian CAN 16-005ND isolate. Interestingly, the two Norwegian 2018 isolates each contained one unique amino acid differences not found in the remaining isolates, V_107_ vs. A_107_ in NOR-2018/SF and D_252_ vs. N_252_ in NOR-2018/NL (Supplementary Table S2). In the remaining five segments (S2, S3, M1, M3, L3) sequence and phylogenetic analysis did not reveal viral sequences linked to the phenotypical differences observed in the challenge trial.

Overall, sequence analysis of the six PRV-1 isolates could not attribute virulence directly to a specific gene segments, viral proteins or amino acid positions. The data suggest that virulence of PRV-1 may be linked to combined involvement and possible gene linkage of genomic segments S1, M2, L1 and L2 and S4 and their encoded proteins.

## 3. Discussion

In this study a dose standardized challenge trial demonstrated that there are virulence differences between PRV-1 isolates in Atlantic salmon regarding induction of histopathological changes typical for HSMI. The high virulent isolates, that is, two Norwegian field isolates (NOR-2018/SF, NOR-2018/NL), induced cardiac lesions consistent with HSMI. The low virulent isolates, that is, three historical Norwegian isolates (NOR-1997, NOR-1996, NOR-1988) and one Canadian isolate (CAN 16-005ND) induced mainly mild cardiac lesions. 

Conflicting results have been reported in the field of PRV-1 research with both success [2,21] and failure [12,13] to produce cardiac lesions consistent with HSMI. Our results suggest that this dichotomy can be at least partly explained by virulence differences. The severe cardiac lesions induced by the Norwegian 2018 field isolates in the present study are consistent with those observed in previous trials using another contemporary Norwegian field isolate (NOR-2012) [2]. In contrast, only mild lesions were induced by the CAN 16-005ND isolate, which is comparable to the lesions observed in a previous trial using two Canadian isolates, including the CAN 16-005ND isolate [12]. Similar low-grade lesions were observed for the historical Norwegian isolates that had been collected prior the emergence of HSMI, which support the hypothesis that PRV-1 isolates present in Norway before HSMI was first recognized in 1999 were of low virulence. However, our results suggest that all PRV-1 isolates can induce cardiac lesions but of different severity.

High loads of PRV-1 in blood cells have previously been well documented after infection with both Norwegian and Canadian isolates [2,12,13]. The present study confirmed this general finding but also demonstrated differences between the virus isolates. For the low virulent isolates, all blood cells were PRV positive with a higher total load of viral protein in blood cells compared to the high virulent isolates in which virus could be detected in only a subpopulation of blood cells. These differences could not be seen by the detection of viral RNA in blood cells. However, the protein detection did not differentiate between intracellular or surface bound virus and targeted a structural protein. Mammalian reoviruses are known to bind sialic acid which is abundant on the erythrocyte surface [22,23]. Combining surface and intracellular staining of blood cells including detection of both structural and non-structural viral proteins, could possibly identify the cell population with replicating virus.

Interestingly, a higher plasma viremia was detected for the high virulent isolates (NOR-2018 isolates) than for the low virulent isolates (historical Norwegian and Canadian isolates). Previous studies of Canadian isolates have not detected PRV-1 in plasma during infection [12] but have been reported using Norwegian isolates [2,11,14]. In our study, virus was detected in plasma at peak of infection in all isolates, including all low virulent isolates but the latter with lower amount as confirmed by detection of viral RNA and multiple viral proteins. The amount of plasma viremia is an important factor with a potential direct effect on pathogenesis and shedding and could be a defining virulence factor. The difference in plasma viremia between high and low virulent isolates could either indicate a more efficient release of high virulent isolates from infected cells or reflect more efficient attachment of low virulent isolates to the erythrocytic surface. Release of virus could be caused by lytic or non-lytic shedding from infected cells or possibly through the process of removal of infected erythrocytes from the circulation, in the spleen. The high amount of virus in plasma following infection with high virulent isolates did not coincide with reduced hemoglobin concentration. This could indicate a non-lytic exit of virus. Although non-enveloped viruses usually cause lysis of the infected cell, non-lytic shedding has recently been characterized for mammalian reovirus [24]. Alternatively, the different levels of plasma viremia could be differences in affinity and binding to erythrocytic surface molecules. Virus’ affinity for and binding to the erythrocyte surface has been known for several virus families, including the reoviruses [25]. The infectious salmon anemia virus is an example of a salmonid virus that binds to the sialic acids on the erythrocyte surface [26]. PRV-1 has a putative conserved sialic binding motif in the σ1 amino acid sequence [16]. Higher affinity to erythrocytes and thus more efficient removal of virus from plasma of the low virulent virus isolates could therefore, theoretically, explaining the differences in plasma viremia between the low- and high virulent groups. This is in accordance with the findings of presence of PRV-1 proteins in all erythrocytes in the low virulent groups but only in a fraction of the erythrocytes in the high virulent groups. Further characterization of the binding to and release from erythrocytes is needed to explain the differences in plasma viremia between high and low virulent isolates. It should be noted that at 6 wpc there was a substantial drop with little or no viral RNA in plasma. This was most pronounced in the Norwegian 2018 and Canadian isolate, thus not consistent with virulence differences. However, the results were surprising and difficult to put into a biological context. Although we did not reveal any quality issues with the samples collected at this timepoint, a non-biological explanation cannot be ruled out.

The ability to induce the heart lesions observed during HSMI, could be correlated to the level of plasma viremia due to more circulating virus available for infection of cardiomyocytes. In the high virulent groups, a high number of PRV positive cardiomyocytes was detected during development of HSMI, consistent with a previous study of a Norwegian field isolate [11]. In comparison, lower amount of virus was detected in cardiomyocytes in the low virulent groups. The results of the in-situ hybridization were supported by the RT-qPCR of viral RNA. The difference in load could therefore be directly correlated to the level of plasma viremia. Alternatively, the high and low virulent isolates differ in their ability to enter or replicate in cardiomyocytes specifically. High loads of PRV in cardiomyocytes of infected fish have been observed in a reported outbreak in Canada [6,27]. It should be noted that the isolate of that outbreak, called B5690, is similar but not identical to the CAN 16-005ND isolate used in the present study [12]. Furthermore, data from field material and a controlled challenge trial are not directly comparable. 

Differences in the innate antiviral response have been reported for PRV-1 infection in Atlantic salmon. Norwegian isolates have been reported to trigger a strong innate response in blood in multiple challenge trial and during ex vivo infection of erythrocytes [2,15,28]. In contrast, infection with Canadian isolates have been reported with little or no immune response [12,13]. In the present study all isolates induced a strong innate antiviral response at similar levels with no differences that could be correlated to virulence. The reason for the previous deviating results is not known but could be attributed to host or technical factors. Furthermore, the differences in viral protein in blood and release of virus to plasma observed in the present study indicates other important virus-host interactions.

Sequence analysis of the high and low virulent isolates identified five segments (S1, M2, L1, L2, S4) which encode viral proteins that could be linked to phenotypical difference. Previous studies have reported evolution of segment S1 and M2 in Norway to be associated with the emergence of HSMI [5], whereas these segments appear to have been relatively stable in isolates from North American Pacific Coast and the Faroe Islands with little or no HSMI [5,29]. In our study, the high virulent NOR-2018 isolates have the evolved variant of S1 and M2, whereas the low virulent Canadian isolate, NOR-1988 and NOR-1996 isolates cluster with the non-evolved variants. However, the NOR-1997 isolate was shown to be of low virulence but have S1 and M2 segments almost identical to that of the NOR-2018s. This excludes the possibility that the S1-M2 alone can explain the virulence differences. Interestingly, NOR-1997 has unique L1 and L2 segments, while these segments were almost identical in the other isolates. An assumed reassortment event combining the S1 and M2 segments of NOR-1997 with L1 and L2 segment of either NOR-1988 or NOR-1996 would in fact generate a virus with a genetic constellation similar to the virulent NOR-2018 isolates.

The segments (S1, M2, L1, L2, S4) and their encoded viral proteins (σ3, µ1, λ2, σ1, λ3 and p13) are potentially related to virulence but the underlying mechanism for development of HSMI were not clarified. The proteins include four structural proteins and two non-structural proteins. Interestingly, the structural proteins are all part of the outer capsid, including σ3 (S1), µ1 (M2), λ2 (L1) and σ1 (S4) [16]. Each protein has specific functions during entry and replication but they are also structurally linked in the outer capsid [30]. The function of the outer capsid proteins is balancing between the capsid stability and optimal attachment and capsid disassembly during cell entry [31]. The increased size and number of salmon farms has substantially increased the number of available hosts. A segmented RNA virus such as PRV will explore the available sequence space through mutation and reassortment to optimize its fitness accordingly. Further investigations are needed to see if the change in outer capsid proteins is related to particle stability, affinity to erythrocytes or replicative potential. 

In virulence investigations, the viral receptor binding protein is often given specific attention. These proteins are major determinants of cell tropism and therefore often also of virulence, which is the case for σ1 in mammalian reovirus (MRV) [32]. In the present study, variation was observed in PRV-1 σ1 (S4). The historical Norwegian and the Canadian isolates had identical σ1 amino acid sequence, whereas both Norwegian 2018 isolates differed from these by one amino acid, which was located at different positions: V107A in NOR-2018/SF and D252N in NOR-2018/NL. If implicated in virulence, these two amino acid changes imposed a common phenotypic effect.

The isolate reported to cause cardiac lesions in field in BC Canada (B5690) has been reported to be similar but not identical to the CAN 16-005ND isolate used in the present study [12]; the isolate contains the non-evolved variant of segment S1 and M2 and lack the unique sequence of NOR-1997 in segment L1 and L2. Interestingly, the Canadian B5690 isolate contains an amino acid difference in σ1 (segment S4) position 252 (D252E) [6]. Furthermore, another Canadian isolate (CAN 16-011D) which failed to induce cardiac lesions in a challenge trial [12] has the same V107A change in σ1 as the virulent NOR2018-SF (data not shown). The latter finding could indicate that this amino acid position is not related to virulence. Overall, the amino acid positions 107 and 252 in the σ1 protein appears to be variable but functional effects of these variations are not clear.

Sequence differences in the two non-structural proteins λ3 (L1) and p13 (S1) were identified comparing high and low virulent isolates. The λ3 protein is the RNA-dependent RNA polymerase and thus key to replication [16]. The λ3 amino acid sequence was almost identical for most isolates, except for the unique sequence observed in NOR-1997 but a potential role in virulence is not obvious. The p13 protein has been shown to be a cytotoxic integral membrane protein [33]. It is encoded by an internal ORF on the bicistronic S1 segment and thus the coding sequences of p13 and σ3 proteins are intrinsically linked. Several of the structural proteins of reoviruses counteract the innate antiviral activity during replication, including the outer structural protein σ3 and λ2 [34]. However, the analysis of the innate response did not indicate any substantial differences between low-and high-virulent isolates.

The present study used a dose standardized challenge model to study virulence. It is important to state that the combination PRV-1 and its natural host Atlantic salmon were the focus of this study. Although the conclusions regarding virulence factors in other salmonid species or of other PRV subtypes cannot be superimposed, the setup could be used in future investigations for different salmonid species and other PRV subtypes. In the trial, fish were challenged with a relatively high dose (5 × 10^7^ particles per fish). Previous dose response trials suggest that high and low dose affect the timing of infection but not the severity of lesions [2]. In the trial we challenged the fish by injection to provide a synchronized infection of fish within the group, which also enabled a timed comparison between different groups. Cohabitation challenge would provide a natural route of infection and add confirmation to the results but a classical cohabitation trial would make synchronized infection between groups more difficult. Previous studies suggest that cohabitational challenge of PRV-1 induce similar or more severe lesions compared to injection challenge [1,2,9]. Alternatively, a bath challenge using standardized dose of purified virus would provide synchronized infection by the natural route of transmission.

This report is the first confirmation of virulence differences between PRV-1 isolates seen as the ability to induce cardiac lesions of different severity in Atlantic salmon. The high virulent isolates were associated with higher plasma viremia during early replication in blood, hypothesized to be associated with the subsequent pathogenic potential in the heart. Multiple genomic segments were identified with sequence variation differentiating high and low virulent isolates. 

## 4. Materials and Methods 

### 4.1. PRV-1 Isolates Used in the Study 

Six PRV-1 isolates were included in the study; two Norwegian isolates from 2018 (NOR-2018/SF, NOR-2018/NL), three historical Norwegian isolates (NOR-1988, NOR-1996, NOR-1997) and one Canadian isolate (CAN 16-005ND). The two Norwegian 2018 isolates were collected from sea sites in the Norwegian counties Sogn og Fjordane (NOR-2018-SF) and Nordland (NOR-2018/NL) in 2018. The selected isolates were considered to be widespread in farmed Atlantic salmon in Norway but were not collected from an HSMI outbreak. The three historical Norwegian isolates NOR-1988, NOR-1996 and NOR-1997 all originated from sea sites in Norway two, three and eleven years prior to the first description of HSMI. NOR-1988 and NOR-1997 have previously been sequenced and had been passaged once through Atlantic salmon [5], whereas NOR-1996 was identified in an archived plasma sample. The Canadian isolate (CAN 16-005ND) had previously been characterized and failed to induce HSMI in two experimental trials in Canada [12,13]. Briefly, the isolate originated from a cohort of healthy Atlantic salmon in Vancouver Island, Canada, with no history of HSMI and had been passaged three times through Atlantic salmon in Canada [12] and once in Norway.

### 4.2. Virus Propagation 

To obtain fresh blood samples with high viral loads enabling viral purification, all six isolates were passaged once through 90 g Atlantic salmon (StofnFiskur Optimal strain). The trial was conducted at VESO Vikan aquatic research facility (Namsos, Norway) and had been approved by the Norwegian Food Safety Authority (NFDA) according to the European Union Directive 2010/63/EU for animal experiments (permit number 11251). The PRV-1 containing material available varied between the isolates and the following inoculums were prepared: NOR-2018/SF and NOR-2018/NL as heparinized blood diluted in Leibovitz’s L15 medium (Life Technologies, Grand Island, NY, USA) (Cq 19.3 and Cq 23.7 respectively), NOR-1988 as purified virus in PBS (Cq 18.1), NOR-1996 as archived plasma sample diluted in L15 medium (Cq 18.7), NOR-1997 and CAN 16-005ND as pelleted blood cells diluted in L15 medium (Cq 19.5 and Cq 22.6 respectively). The inoculums were administered as intraperitoneal (Norwegian 2018 isolates) or intramuscular (historical Norwegian and Canadian isolates) injection in 16 naïve fish per group, using 0.1 mL per fish. The fish were sampled weekly up to 5 wpc collecting heparinized blood samples which were centrifuged to obtain pelleted blood cells. The load of viral RNA and viral protein was monitored by RT-qPCR and flow cytometry respectively (details described below). One blood pellet sample with high viral load was selected for viral purification: NOR-2018/SF Cq 15.3, NOR-2018/NL Cq 15.7, NOR-1988 Cq 17.9, NOR-1996 Cq 15.7, NOR-1997 Cq 15.4 and CAN 16-005ND Cq 19.6. Complete data set of the propagation shown in Supplementary Table S3.

### 4.3. Virus Purification

The selected blood pellet from each isolate (0.5 mL) was diluted 1:10 in homogenization buffer (10 mM TrisCl, 50 mM NaCl, 10 mM 2-mercaptoethanol) and sonicated on ice for 10 s at 25 kHz five times with 1 min rest in between. PRV-1 was purified from the blood homogenate and viral particles separated on a CsCl gradient as previously described [2]. The bottom of the tube was punctured using a 21G needle, fractions collected by gravitational drop (0.5–1.0 mL fractions) and density determined by cross referencing the refractive index figure with those computed by Bruner and Vinograd, 1965 [35]. For each isolate, the CsCl fraction with the buoyant density of PRV-1 (1.34 g/mL) [2]) was injected into a Slide-A-Lyzer Dialysis Cassette (Thermo Fisher Scientific, Waltham, MA, USA) and dialyzed against Dulbecco’s PBS without calcium and magnesium (Sigma-Aldrich, St. Louis, MO, USA) for >1 h, >3 h and then >12 h. PRV-1 particles were verified in all batches by transmission electron microscopy (TEM) of negative stained samples as previously described [2]. A filamentous material was also observed by TEM, it could not be identified and was similar in all batches of virus. The purified batches were stored with 15% glycerol at −80 °C until used.

### 4.4. Virus Quantification

The copy number of the bathes of purified virus was determined by absolute quantification RT-qPCR. RNA was isolated from a 10 μL sample, diluted in 130 μL Dulbecco’s phosphate buffered saline (DPBS), using QIAamp Viral RNA mini kit according to manufacturer’s instructions (Qiagen, Hilden, Germany), eluting RNA in 50 μL. The eluted template RNA was denatured at 95 °C for 5 min using 5 μL as input for the RT-qPCR reaction performed with Qiagen OneStep kit (Qiagen, Hilden, Germany) targeting PRV segment S1, run with triplicate samples (conditions described later under PRV RT-qPCR). Absolute quantification of the PRV-1 particles was performed using a standard curve made from in vitro transcripts of the PRV segment S1 open reading frame (ORF) prepared as previously described [2]. An eight-point standard curve from 10-fold serial dilutions of transcripts, ranging from 10^8^ to 10^1^ transcripts per sample was run with the purified PRV batches. The batches of purified virus contained the following copy number: NOR-2018/SF 7.3 × 10^9^ copies/mL, NOR-2018/NL 2.4 × 10^10^ copies/mL, NOR-1988 4.0 × 10^9^ copies/mL, NOR-1996 1.6 × 10^10^ copies/mL, NOR-1997 8.1 × 10^10^ copies/mL, CAN 16-005ND 2.8 × 10^9^ copies/mL.

### 4.5. Sequencing and Genome Assembly

RNA was isolated from 200 μL of the batches of purified virus from all six isolates (except 100 μL for CAN 16-005ND) using a combination of Trizol LS (Life Technologies) and RNeasy Mini spin column (Qiagen, Hilden, Germany). In brief, the purified virus was mixed with Trizol LS, added chloroform, then separating the phases by centrifugation. The aqueous phase was collected and proceeded with the RNeasy Mini spin column (Qiagen, Hilden, Germany) as recommended by the manufacturer, eluting isolated RNA in 30 μL RNase-free water.

Next-Generation Sequencing was performed using the Ion Total RNA-Seq Kit v2 library preparation kits (Thermo Fisher Scientific) following the manufacturer’s recommendations. The library preparation was setup up on a Ion Chef system and the samples were sequenced on Ion S5™ System from (Thermo Fisher Scientific). 

The sequence reads generated by next-generation sequencing were imported to CLC genomics workbench v10.0.1 (Qiagen, Hilden, Germany) and mapped to reference genome, Piscine orthoreovirus isolate NOR2012-V3621. Default mapping parameters, mismatch cost = 2, insertion cost = 3 and deletion cost = 3 were used. Consensus sequences were generated from the mapping for all the individual segments of the PRV-1 isolates. Total number of mapped reads and average coverage mapping of PRV isolates is shown in Supplementary Table S4. A list of the PRV-1 isolates used in the present study and their accession numbers are shown in Table 1.

### 4.6. Sequence and Phylogentic Analysis

Multiple sequence alignments were performed using MUSCLE [36] and MEGA X software v10.1.7 (available from www.megasoftware.net) [37]. Phylogenetic analyses were performed with MEGA X using full-length sequences from all ten gene segments and the eleven proteins known to be encoded by PRV-1. Maximum likelihood (ML) was used to generate the phylogenetic trees from gene segment sequences and neighbor joining (NJ) for the amino acid sequences, using the best-fitting substitution models suggested by the program. PRV-3 NOR/060214 (MG253807–MG253816) was selected as outgroup in all trees. Bootstrap values were calculated from 1000 replicates and values above 70 were considered significant [20].

### 4.7. Experimental Challenge Trial

An experimental challenge trial comparing the six PRV-1 isolates was conducted at VESO Vikan aquatic research facility (Namsos, Norway). The trial had been approved by the Norwegian Food Safety Authority (#11251) according to the European Union Directive 2010/63/EU for animal experiments. A total of 500 Atlantic salmon (StofnFiskur Optimal strain) with an initial average weight of 90 g was used. The fish population had been screened (10 fish) and found negative for ISAV, SAV, PRV-1, PMCV and IPNV by RT-qPCR. Prior to challenge, the fish were acclimatized for one week. The fish were kept in tanks supplied with seawater of 32‰ salinity (30–36‰) at 12 ± 1 °C with continuous light, fed according to standard procedures and observed minimum once per day. The fish were anesthetized by bath immersion in benzocaine chloride (2 ± 5 min, 0.5 g/10 L water) prior to handling and euthanized using a higher concentration of benzocaine chloride (1 g/5 L water). 

The experiment included six groups with the PRV-1 isolates NOR-2018/SF, NOR-2018/NL, NOR-1988, NOR-1996, NOR-1997, CAN 16-005ND and one negative control group. In each group, 70 fish were challenge with an intramuscular injection of purified virus in PBS (100 uL), using a standardized dose of 5 × 10^7^ particles per fish. The control group was injected with PBS.

At 1, 2, 3, 4, 6, 8 and 10 wpc, 10 fish were sampled from each group. Heparinized blood was collected from the caudal vein and the hemoglobin concentrations was immediately measured by HemoCue Hb 201+ (HemoCue, Ängelhom, Sweden) according to the manufacturer’s instructions. The blood cells and plasma were analyzed for PRV protein by flow cytometry and western blotting respectively. Blood was centrifuged at 3000 *g* for 5 min at 4 °C to separate blood cells and plasma which was analyzed for PRV RNA content by RT-qPCR. The blood cells and plasma were analyzed for PRV protein by flow cytometry and western blotting respectively. Tissue from a heart was sampled in RNAlater (Life Technologies, Carlsbad, CA, USA) for RT-qPCR analysis and parallel samples were harvested in 10% phosphate buffered formalin and used for histological analysis and in-situ hybridization.

### 4.8. Detection of Viral Protein by Flow Cytometry

Blood cells were analyzed for PRV-1 σ1-protein by flow cytometry. All steps were performed on ice. Heparinized blood were diluted 1:20 in flow buffer (PBS+ 1% BSA+ 0.05% azide), plated in 50 μL aliquots into 96-well plates and washed in flow buffer. The cells were fixed in IC Fixation Buffer (eBioscience, San Diago, CA, USA) and washed in Permeabilization Buffer (eBioscience, San Diago, CA, USA). The blood cells were stained for 30 min using polyclonal antibodies raised against PRV-1 σ1 with a 1:5000 dilution (Anti-σ1, #K275) [9]. Finally, the cells were stained with secondary Alexa Fluor 488 conjugated anti-rabbit IgG (Molecular Probes, Eugene, Oregon, USA) (2 mg/mL diluted 1:800) for 30 min. As a negative control, parallel samples were run using the same procedure but without primary antibody. The stained samples were read on a Gallios Flow Cytometer (Beckman Coulter, Indianapolis, IN, USA) counting 30,000 cells per sample and the data were analyzed using the Kaluza software (Beckman Coulter). For each sample, the ΔMFI was calculated by subtracting the MFI of the negative control (without primary antibody) from the MFI of the sample with primary antibody.

### 4.9. RNA Isolation

From pelleted blood cells, total RNA was isolated from 20 μL samples, which was homogenized in QIAzol Lysis Reagent (Qiagen, Hilden, Germany) using 5 mm steel beads and TissueLyser II (Qiagen, Hilden, Germany) for 2 × 5 min at 25 Hz. Then added chloroform, centrifuged, collected the aqueous phase and proceeded with automated RNA isolation with the RNeasy Mini QIAcube Kit (Qiagen, Hilden, Germany) as described by the manufacturer. From heart samples, total RNA was isolated from 25 mg samples using RNeasy Mini QIAcube Kit (Qiagen, Hilden, Germany) as described by the manufacturer. The RNA was quantified using a NanoDrop ND-1000 spectrophotometer (Thermo Fisher Scientific).

For plasma samples, total RNA was isolated from a 50 μL sample, diluted in 90 μL PBS, using QIAamp Viral RNA mini kit according to manufacturer’s instructions (Qiagen, Hilden, Germany) and eluting RNA in 50 μL.

### 4.10. Detection of Viral RNA by qPCR

Detection of PRV-1 RNA from blood cells, heart and plasma was performed by RT-qPCR targeting segment S1 using previously described primers and probe [2]. For heart and blood cell samples, input RNA was set to 100 ng (5 μL of 20 ng/μL) of total RNA which had been denatured at 95 °C for 5 min. The RT-qPCR was performed using Brilliant III Ultra-Fast QRT-PCR Master Mix (Agilent, Santa Clara, CA, USA) according to the manufacturer’s instruction with 400 nM primer and 300 nM probe. The following cycle parameters was used: 10 min at 50 °C, 3 min at 95 °C, 35 cycles of 95 °C/5 s and 60 °C/ 10 s run in AriaMx (Agilent, Santa Clara, CA, USA). For the plasma samples, input RNA was set to 5 μL out of the 50 μL eluted RNA which had been denatured at 95 °C for 5 min. The RT-qPCR was performed using the Qiagen OneStep kit (Qiagen, Hilden, Germany) with the following conditions: 400 nM primer, 300 nM probe, 400 nM dNTPs, 1.26 mM MgCl2 and 1 × ROX reference dye. The following cycle parameters was used: 30 min at 50 °C, 15 min at 95 °C, 35 cycles of 94 °C/15 s, 60 °C/30 s and 72 °C/30 s in AriaMx (Agilent, Santa Clara, CA, USA). All samples from blood cells, heart and plasma were run in duplicate and a sample was defined as positive if both parallel samples had a Ct < 35.

### 4.11. Detection of Viral Protein by Western Blotting 

Western blotting was performed to compare loads of PRV proteins in plasma (0.4 μL) at 3 and 4 wpc. The proteins were separated by sodium dodecyl sulfate-polyacrylamide gel electrophoresis (SDS-PAGE), using 4–12% Bis-Tris Criterion XT PreCast gels (Bio-Rad, Hercules, CA, USA). The proteins were transferred to a polyvinylidene fluoride membrane (Trans-Blot Rurbo Midi PVDF, Bio-Rad, Hercules, CA, USA) and incubated with primary antibody overnight at 4 °C using the following sera diluted 1:500; Anti-σ1 #K275 [9], Anti-μ1C #K265 [9], Anti-λ1 #K273 [38] and Anti-σNS 01BO [39]. After incubation with secondary antibody, anti-rabbit IgG-HRP (Amersham, GE Healthcare, Buchinghamshire, UK) (1:50,000), the PRV proteins were detected by chemiluminescence (Clarity Western ECL Substrate, Bio-Rad, Hercules, CA, USA). MagicMark (XP Western Protein Standard, Invitrogen) was used as ladder. The western blot was performed on plasma samples at 3 and 4 wpc, detecting σ1 in all groups and expanded to include μ1C, λ1 and σNS in group NOR-2018/SF and CAN 16-005ND.

### 4.12. Heart Histopathology Evaluation

Formalin fixed and paraffin embedded sections of heart tissue from the challenge experiment were stained with hematoxylin-eosin (HE). The slides from 3–10 wpc, including all six PRV-1 groups and the negative control group, were blinded for histopathological examination (*n* = 350) and evaluated by one investigator. The scoring was determined by a visual analog scale (0 to 3) based on previously published criteria [2], scoring each heart compartment separately (epicardium, compactum, spongiosum and atrium). The arithmetic mean was calculated for a total cardiac score.

### 4.13. In-Situ Hybridization

To compare the virus RNA load in the heart, heart sections from group NOR-2018/SF, NOR-1988 and CAN 16-005ND sampled at 4, 6 and 8 wpc were stained for PRV RNA by in-situ hybridization using RNAscope^®^ (RED) 2.5 HD Detection Kit (Advanced Cell Diagnostic, Newark, CA, USA) according to manufacturer’s instructions as previously described [40]. In brief, the paraffin embedded tissue sections (5 µm) were dewaxed in ACD HybEZ™ II followed by hydrogen peroxide treatment, then boiled in RNAscope Target Retrieval Reagent and incubated with RNAscope Protease Plus in HybEZ™ oven. Each section was hybridized by RNAscope probe targeting PRV-1 genome segment L3 (Advanced Cell Diagnostics catalog number-537451) [40]. Probe targeting Peptidylpropyl Isomerase B (PPIB) in Atlantic salmon (Advanced Cell Diagnostics, catalog number-494421) was used as reference target gene expression to test for RNA integrity in the samples. As the negative control, probe-DapB (Advanced Cell Diagnostics catalog number-310043) was used to evaluate cross reactivity. Fast Red chromogenic substrate was used for detection of signals amplified following manufacturer’s instructions. Counterstaining was done with 50% Gill’s hematoxylin solution and mounted with EcoMount (BioCare Medical, Pacheco, CA, USA).

### 4.14. Innate Immne Response

The gene expression of antiviral immune genes, including type I interferon (INFab), interferon-stimulated gene 15 (ISG 15) and myxovirus resistance GTPase (Mx1) in blood cells were analyzed at 1–6 wpc for all six PRV-1 groups and the control group. From each sample, 400 ng total RNA was reverse transcribed to cDNA using the QuantiTect Reverse Transcription Kit with gDNA wipeout buffer (Qiagen, Hilden, Germany). For qPCR, cDNA corresponding to 5 ng RNA was analyzed with Sso Advanced Universal SYBR Green Supermix (Bio-Rad, Hercules, CA, USA) and 10 µM of forward and reverse target-specific primers in a 10 µl volume in duplicate wells on a 384 well plate. Probes and primer sequences are given in Supplementary Table S5. The amplification program (95 °C/15 s denaturation, 60 °C/30 s amplification) was run for 40 cycles in a CFX Touch Real-Time PCR Detection System (Bio-Rad, Hercules, CA, USA), followed by a melt point analysis. Results were analyzed using the software CFX Manager, version 3.1.1621.0826. The expression cycle threshold level was normalized against the elongation factor (EF) 1α reference gene (ΔCt) The ΔΔCt method was used to calculate relative expression levels and fold induction compared to samples from the uninfected control group from the same sampling point.

### 4.15. Statistical Analysis

Statistical comparison between different groups was performed using the non-parametric Mann-Whitney test due to the small sample size (*n* = 10). The comparison included PRV RNA load measured as Ct-values (blood cells, plasma and heart), hemoglobin concentration obtained by HemoCue, protein load in blood cells observed as MFI value by flow cytometry, histopathological heart scores and expression on innate immune genes. *p*-values ≤ 0.05 were considered as significant. All statistical analysis described were performed with GraphPad Prism (GraphPad Software Inc., La Jolla, CA, USA).

## 5. Patents

There is a patent pending for genetic markers of PRV-1 virulence-based results generated in the present and previous studies (NMBU and PatoGen).

## Figures and Tables

**Table 1:** List of PRV-1 isolates used in the present study and their accession numbers.

PRV-1 Isolate	Origin	Accession Number
NOR-2018/SF	Norway	MW260135–MW260144
NOR-2018/NL	Norway	MW260145–MW260154
NOR-1997	Norway	MW260155, MW260156, MW279843–279850
NOR-1996	Norway	MW279851–MW279860
NOR-1988	Norway	MW279861–MW279870
CAN 16-005ND	Canada	MW279871–MW279880

## Supplementary Materials

The following are available online at https://www.mdpi.com/2076-0817/9/12/1050/s1, Figure S1. PRV RNA in blood cells; Figure S2. Detection of viral proteins in plasma; Figure S3. Heart histopathology of each heart compartment; Figure S4. Heart lesions NOR-2018/SF; Figure S5. Heart lesions NOR-2018/NL; Figure S6. Heart lesions NOR-1997; Figure S7. Heart lesions NOR-1996; Figure S8. Heart lesions NOR-1988; Figure S9. Heart lesions CAN 16-005ND; Figure S10. PRV positive blood cells in the heart at 4 wpc; Figure S11. Phylogenetic trees from all ten segments for the six PRV-1 isolates; Table S1. Statistical comparison of PRV RNA in plasma; Table S2. Differences in amino acid sequence between the six isolates; Table S3. Viral load in blood cells during PRV-1 propagation; Table S4. Total number of mapped reads and average coverage mapping of PRV-1 isolates; Table S5. Primers for immune gene analyses. File S1. PRV virulence—All data.
