# Supplementary material for: Piscine Orthoreovirus-1 Isolates Differ in Their Ability to Induce Heart and Skeletal Muscle Inflammation in Atlantic Salmon (Salmo salar)
Source: Pathogens. 2020 Dec 14;9(12):1050. doi: 10.3390/pathogens9121050 (PMC7765100; doi:10.3390/pathogens9121050)
Supplement: Supplementary file 1 [file pathogens-09-01050-s001.zip › pathogens-1028286-supplementary/Virulence PRV - Supplementary 20201214.docx]

Supplementary material

Piscine orthoreovirus-1 isolates differ in their ability to induce heart and skeletal muscle inflammation in Atlantic salmon

Øystein Wessel, Elisabeth F. Hansen, Maria K. Dahle, Marta Alarcon, Nina A. Vatne, Ingvild B. Nyman, Karen Bækken Soleim, Dhamotharan Kannimuthu, Gerrit Timmerhaus, Turhan Markussen, Morten Lund, Håvard Aanes, Magnus Devold, Makoto Inami, Marie Løvoll, Espen Rimstad

OVERVIEW

**Figure S1.** PRV RNA in blood cells

**Figure S2.** Detection of viral proteins in plasma

**Figure S3.** Heart histopathology of each heart compartment.

**Figure S4.** Heart lesions NOR-2018/SF

**Figure S5.** Heart lesions NOR-2018/NL

**Figure S6.** Heart lesions NOR-1997

**Figure S7.** Heart lesions NOR-1996

**Figure S8.** Heart lesions NOR-1988

**Figure S9.** Heart lesions CAN 16-005ND

**Figure S10.** PRV positive blood cells in the heart at 4 wpc.

**Figure S11.** Phylogenetic trees from all ten segments for the six PRV-1 isolates

**Table S1.** Statistical comparison of PRV RNA in plasma

**Table S2.** Differences in amino acid sequence between the six isolates

**Table S3.** Viral load in blood cells during PRV-1 propagation

**Table S4**. Total number of mapped reads and average coverage mapping of PRV-1 isolates

**Table S5.** Primers for immune gene analyses


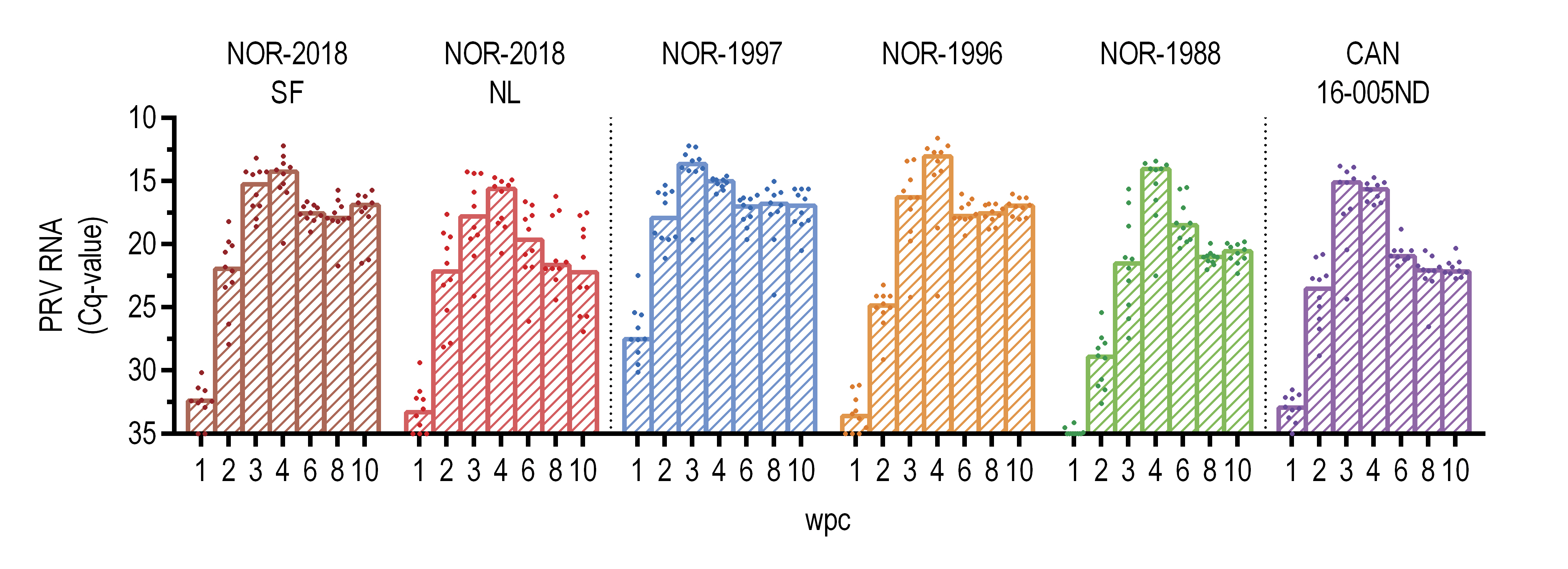


**Figure S1. PRV RNA in blood cells.** PRV RNA in blood cells measured by RT-qPCR, shown as individual and median Cq-values from 1 to 10 weeks post challenge (wpc)


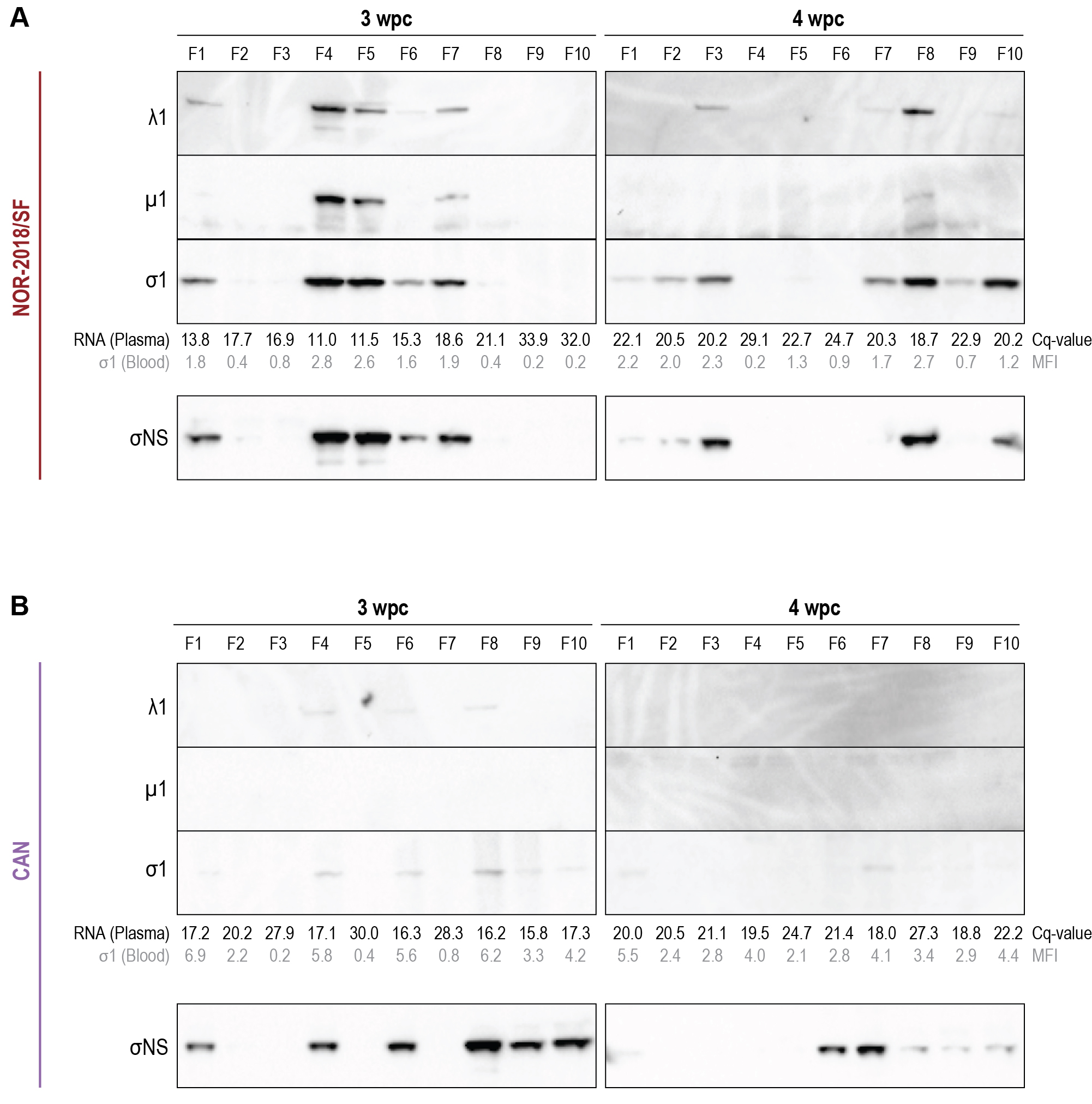


**Figure S2. Detection of viral proteins in plasma.** Detection of structural protein λ1, μ1 and σNS in plasma by Western Blotting at 3 and 4 wpc (n=10) from the groups infected with NOR-2018/SF and the Canadian isolate 16-005ND. For comparison, detection of σ1 from Figure 3B was included. The individual RNA load in plasma (Cq-value) and σ1-protein load in blood cells (MFI) are listed for each fish.


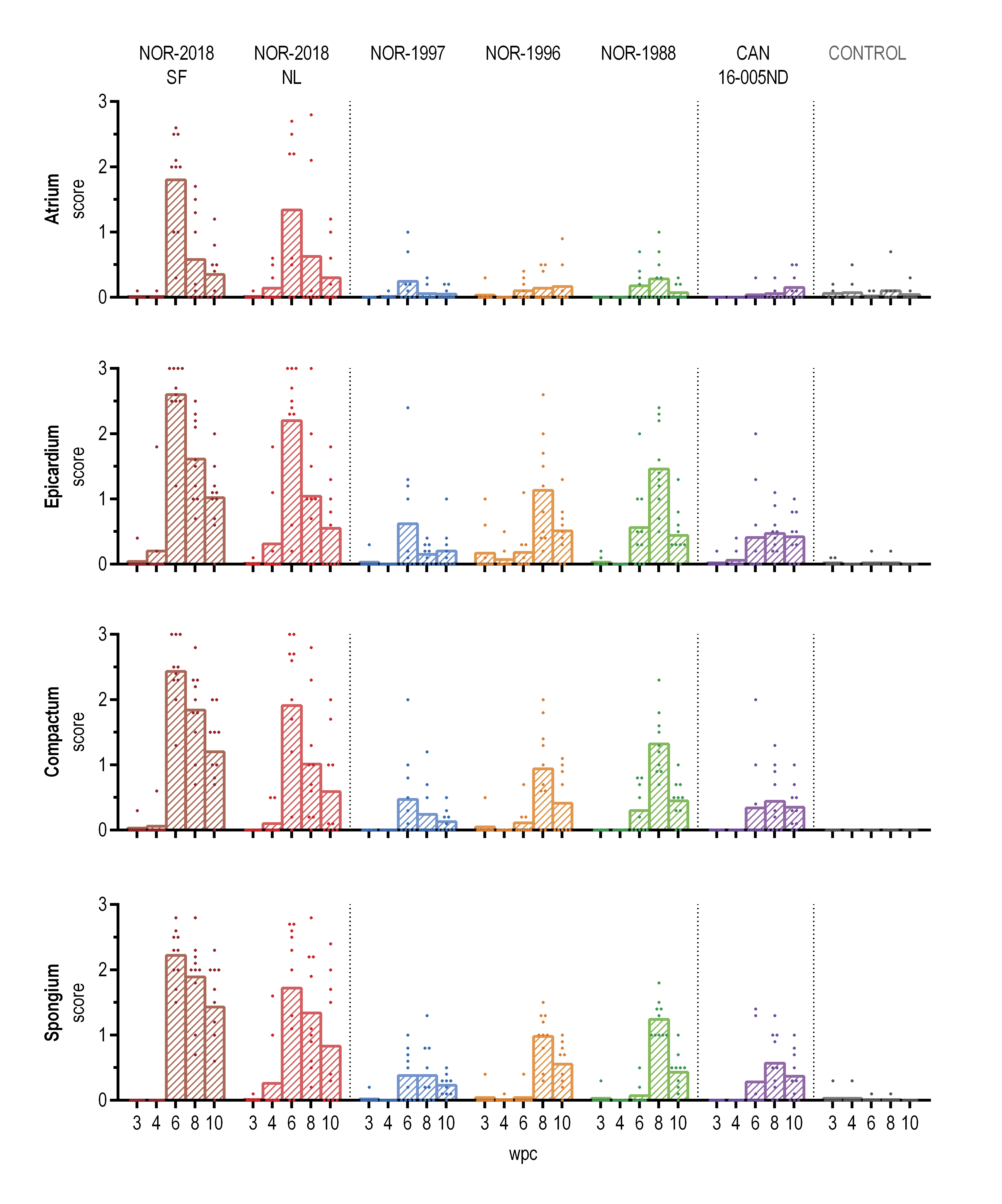


**Figure S3. Heart histopathology of each heart compartment.** Scoring of each heart compartment, including atrium, epicardium, compactum and spongiosum, shown as individual score and group mean from 3 to 10 weeks post challenge (wpc) for the six different PRV groups NOR-2018/SF, NOR-2018/NL, NOR-1997, NOR-1996, NOR-1988 and CAN 16-005ND (color coded) and the control group (grey) (n = 10).

**Figure S4. Heart lesions NOR-2018/NL.** Histopathological images of heart at peak of cardiac lesions (6 wpc, F1-10) after infection with NOR-2018/NL. Total cardiac score in bottom right.**Figure S5. Heart lesions NOR-2018/SF.** Histopathological images of heart at peak of cardiac lesions (6 wpc, F1-10) after infection with NOR-2018/SF. Total cardiac score in bottom right.**Figure S6. Heart lesions NOR-1997.** Histopathological images of heart at peak of cardiac lesions (8 wpc, F1-10) after infection with NOR-1997. Total cardiac score in bottom right.**Figure S7. Heart lesions NOR-1996.** Histopathological images of heart at peak of cardiac lesions (8 wpc, F1-10) after infection with NOR-1996. Total cardiac score in bottom right.**Figure S8.** **Heart lesions NOR-1988.** Histopathological images of heart at peak of cardiac lesions (8 wpc, F1-10) after infection with NOR-1988. Total cardiac score in bottom right.**Figure S9. Heart lesions Can 16-005ND.** Histopathological images of heart at peak of cardiac lesions (8 wpc, F1-10) after infection with CAN 16-005ND. Total cardiac score in bottom right.


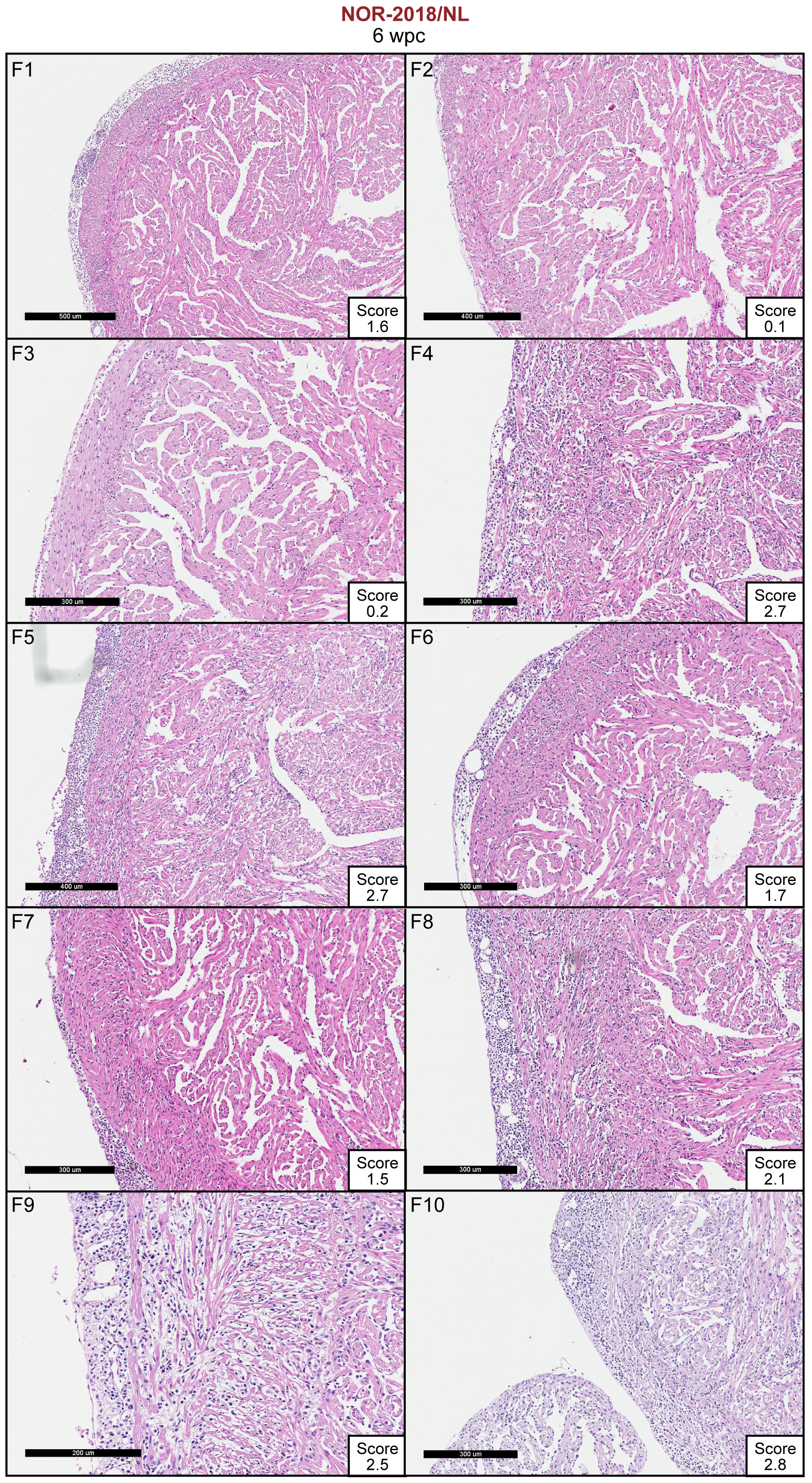

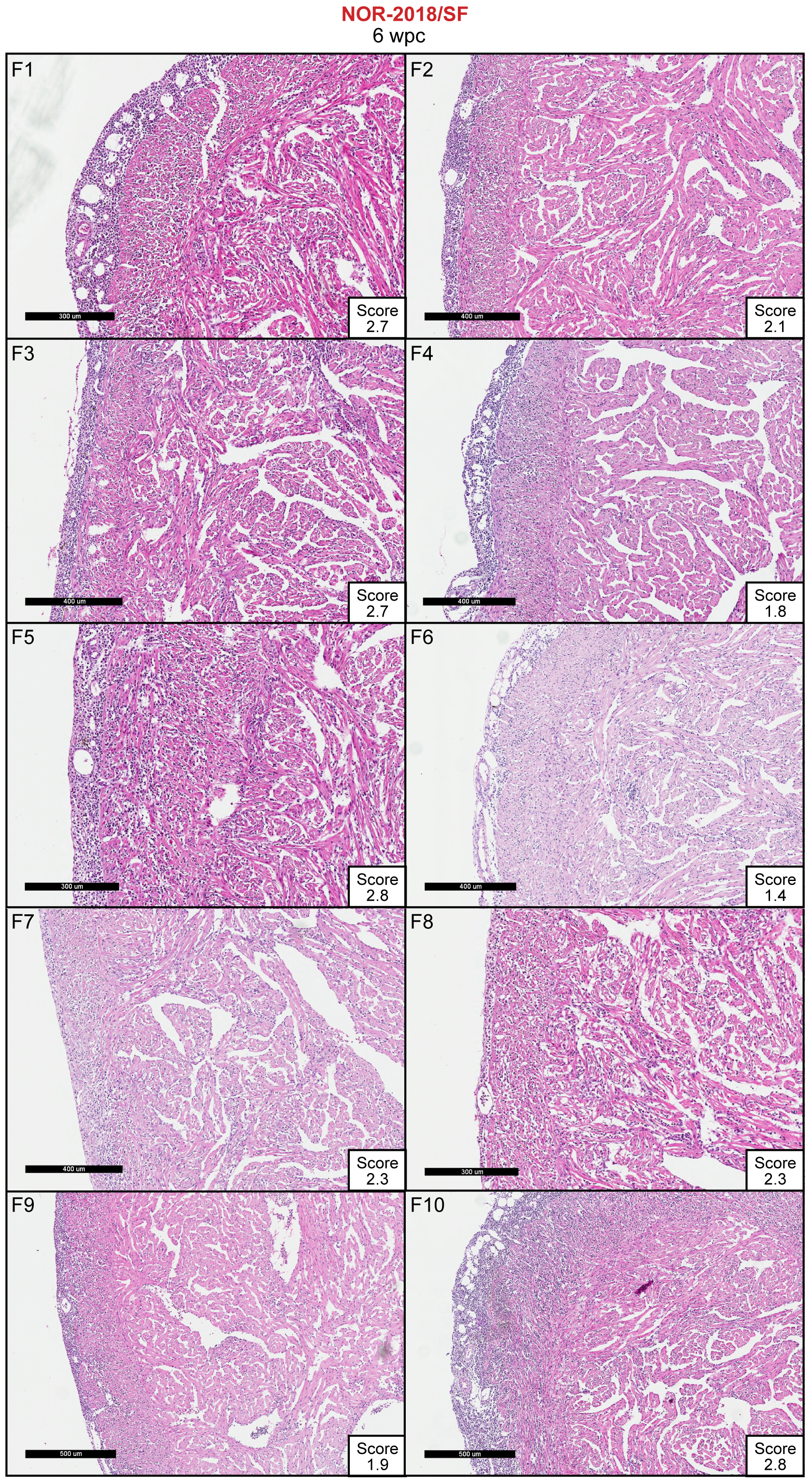

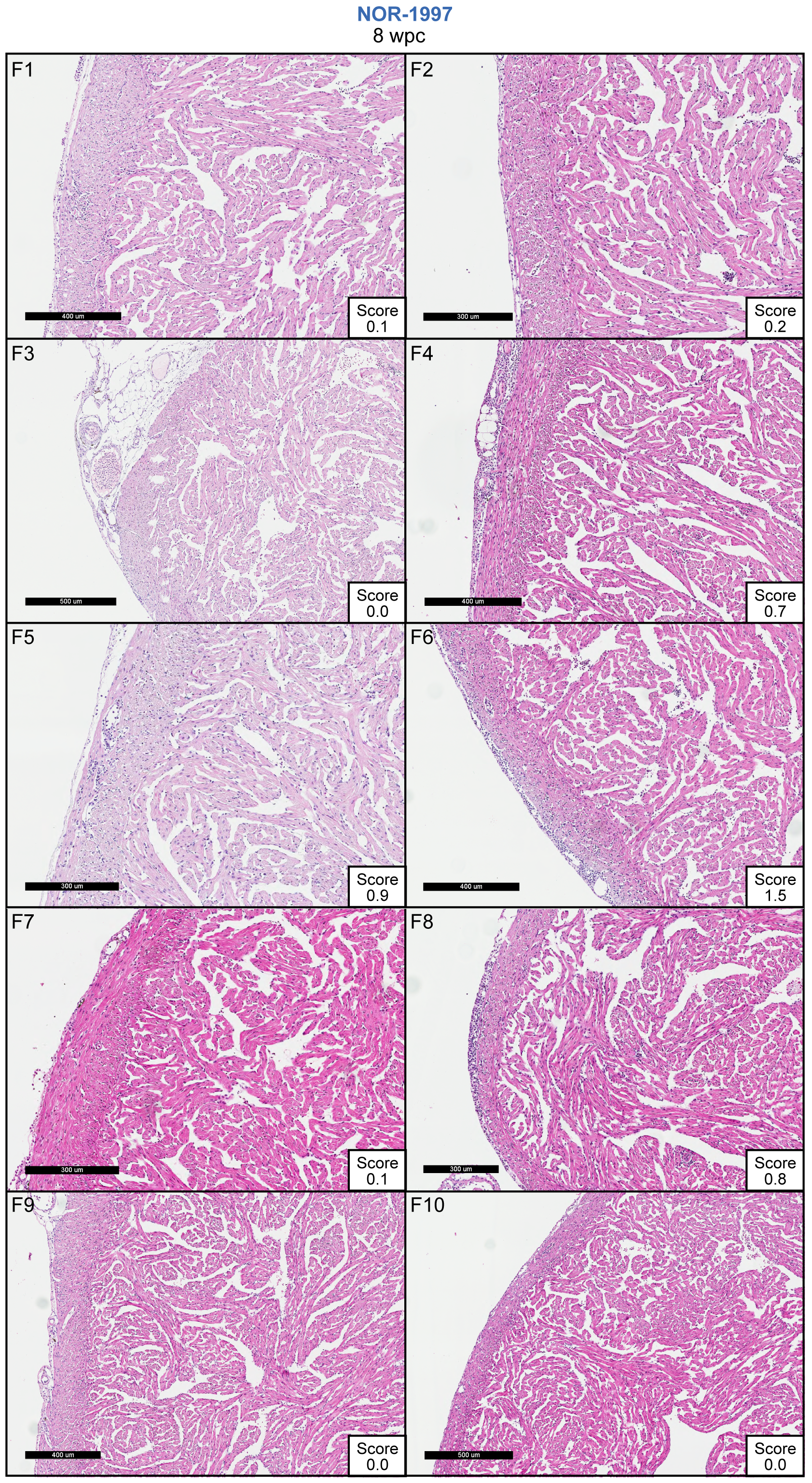

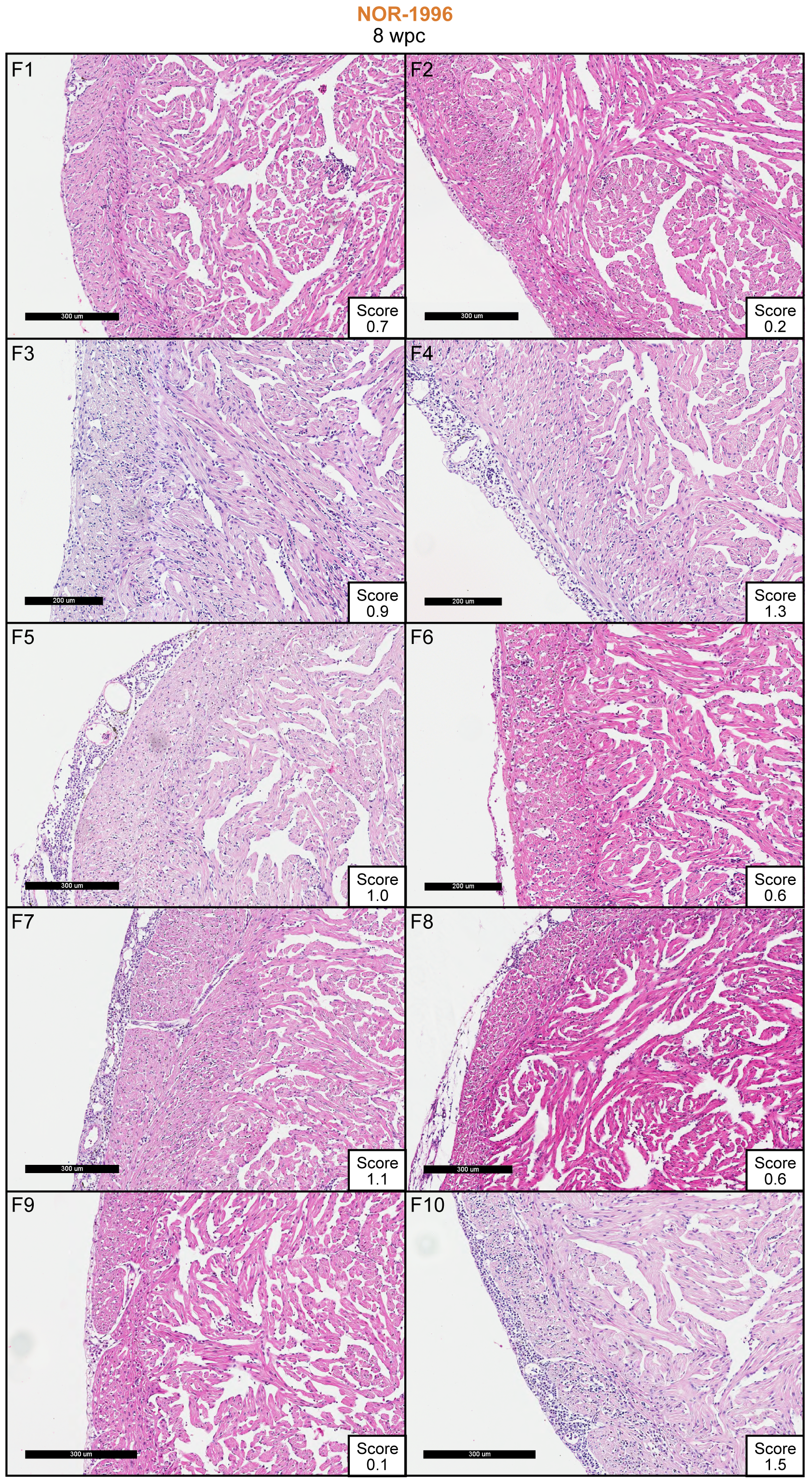

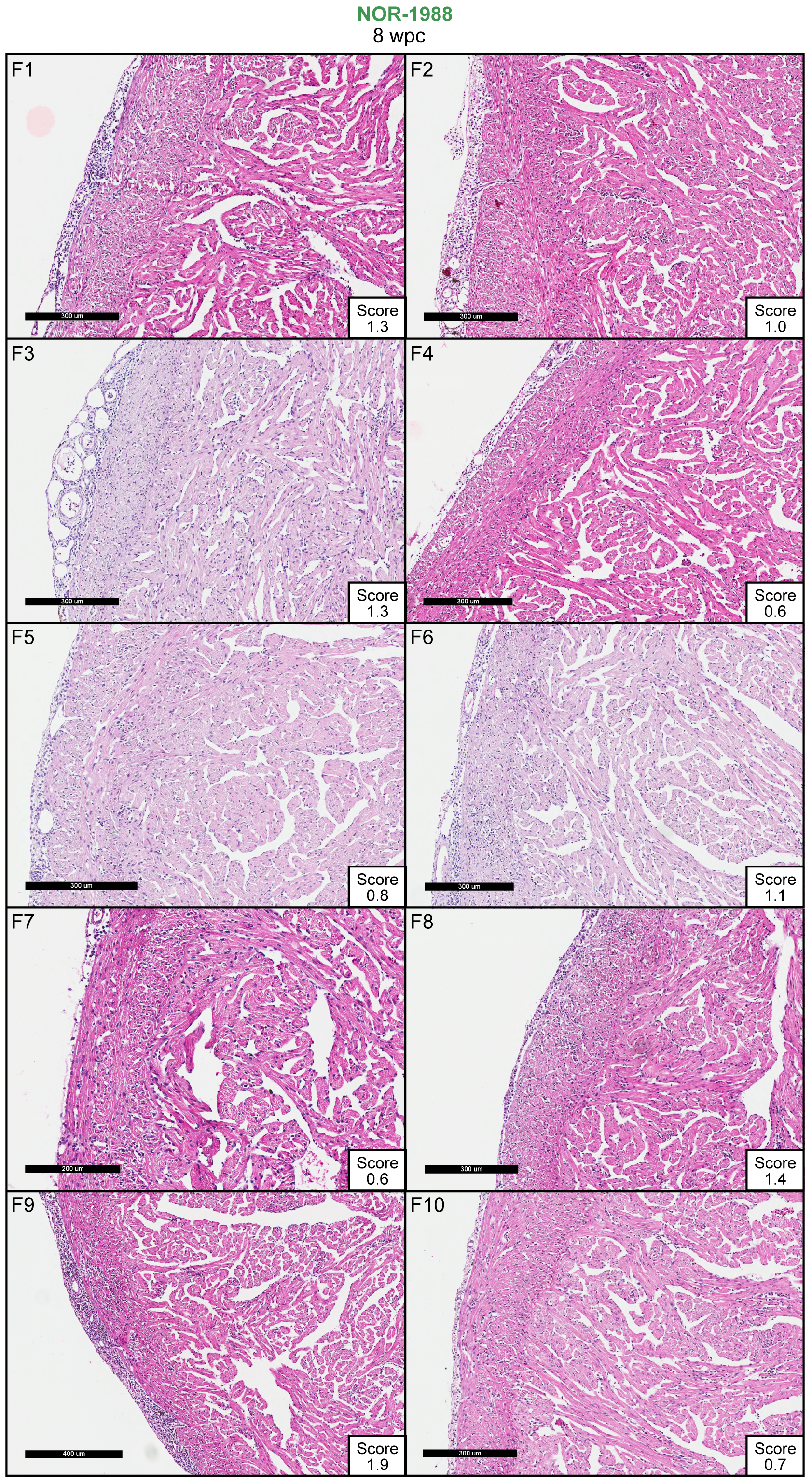

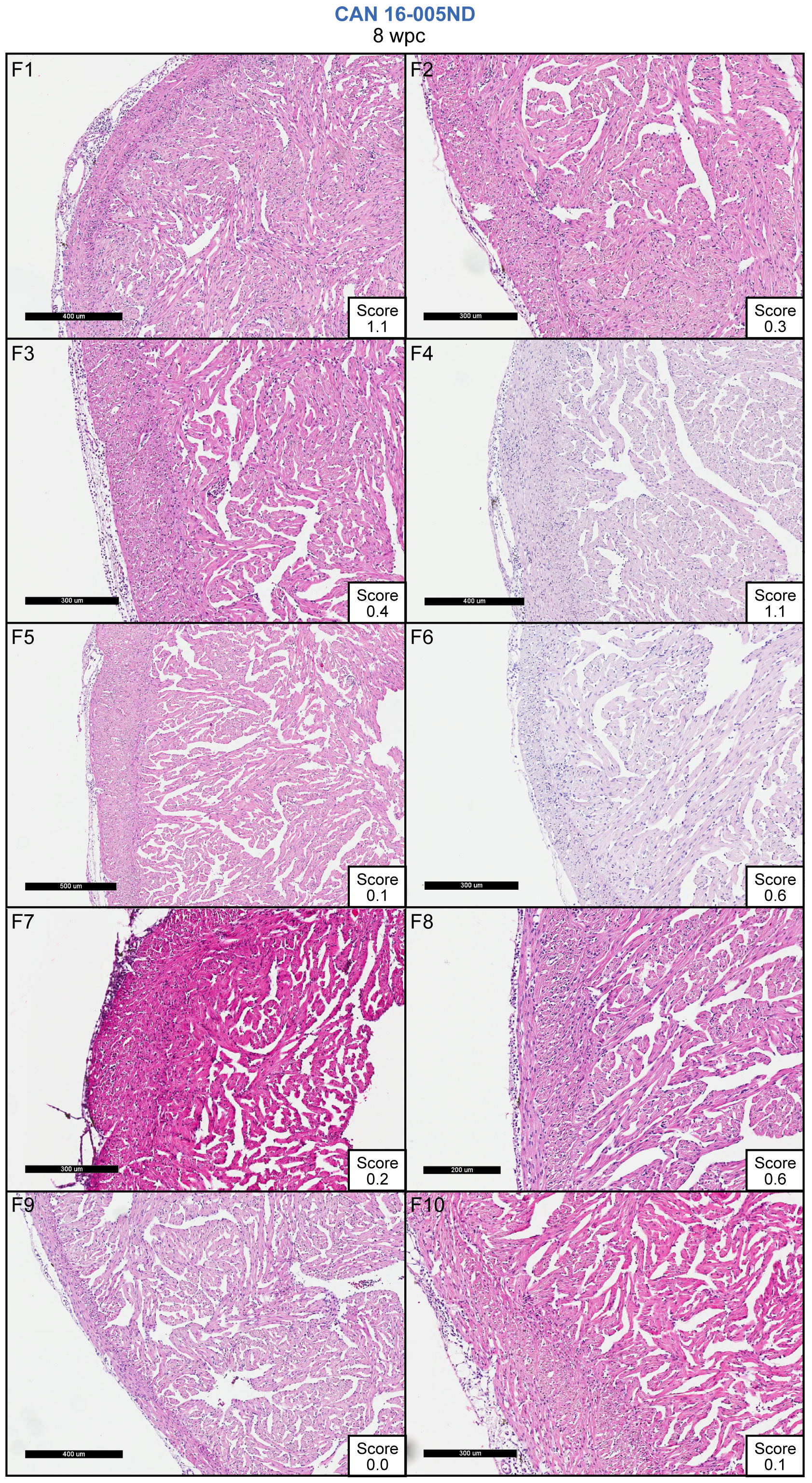

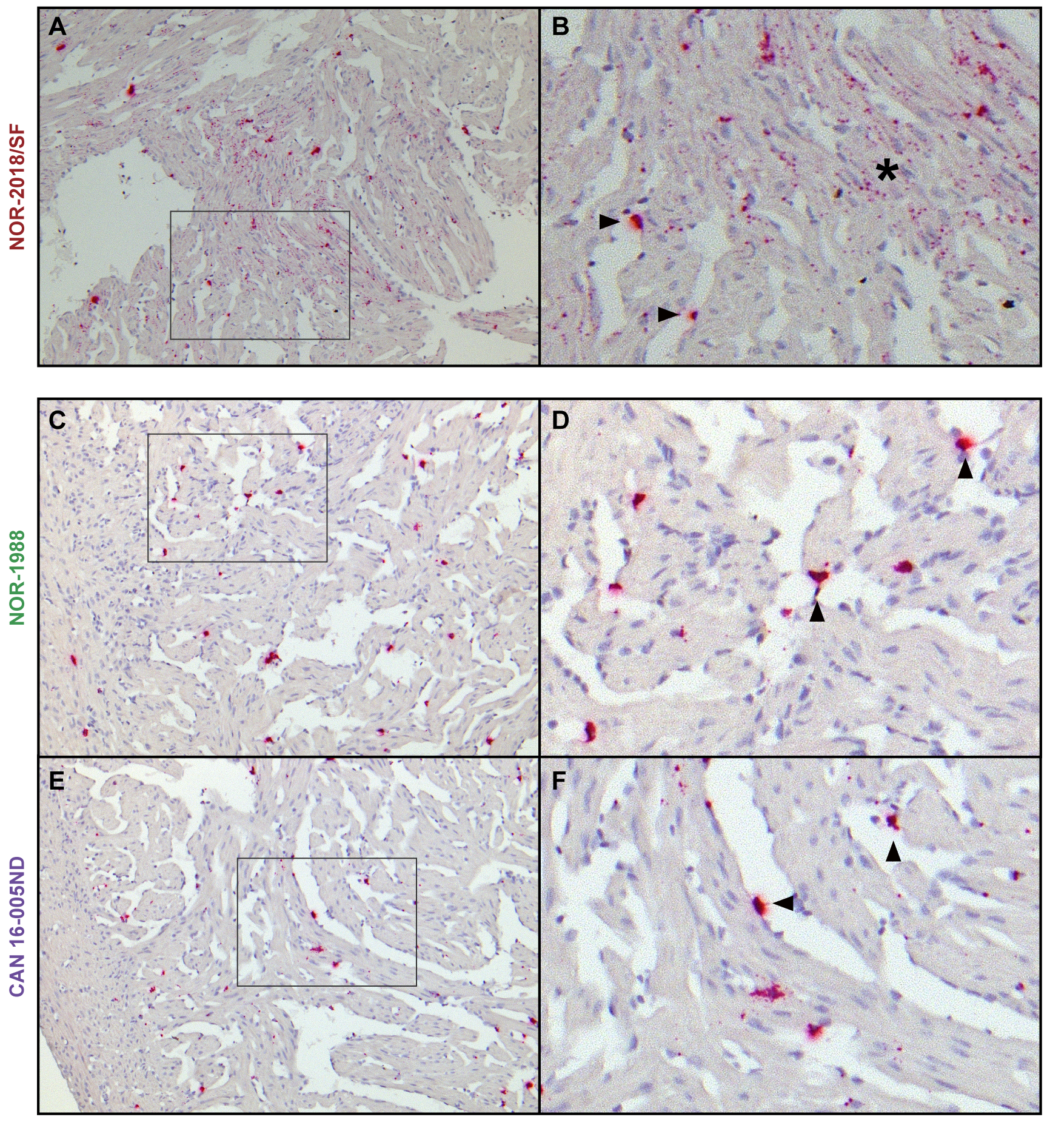


**Figure S10.** PRV positive blood cells in the heart at 4 wpc. Detection of PRV RNA in heart sections performed by *in-situ* hybridization demonstrated numerous positive blood cells at 4 wpc after infection with NOR-2018/SF (A, B). NOR-1988 (C, D) and CAN 16-005ND (E, F). Frame in left panel indicate enlarged area shown in the right panel. Positive staining observed as red staining. Many of the positive blood cells appeared to be attached to the endothelium (arrowheads). In the NOR-2018/SF group (A, B) punctuated staining in cardiomyocytes was also observed (*).


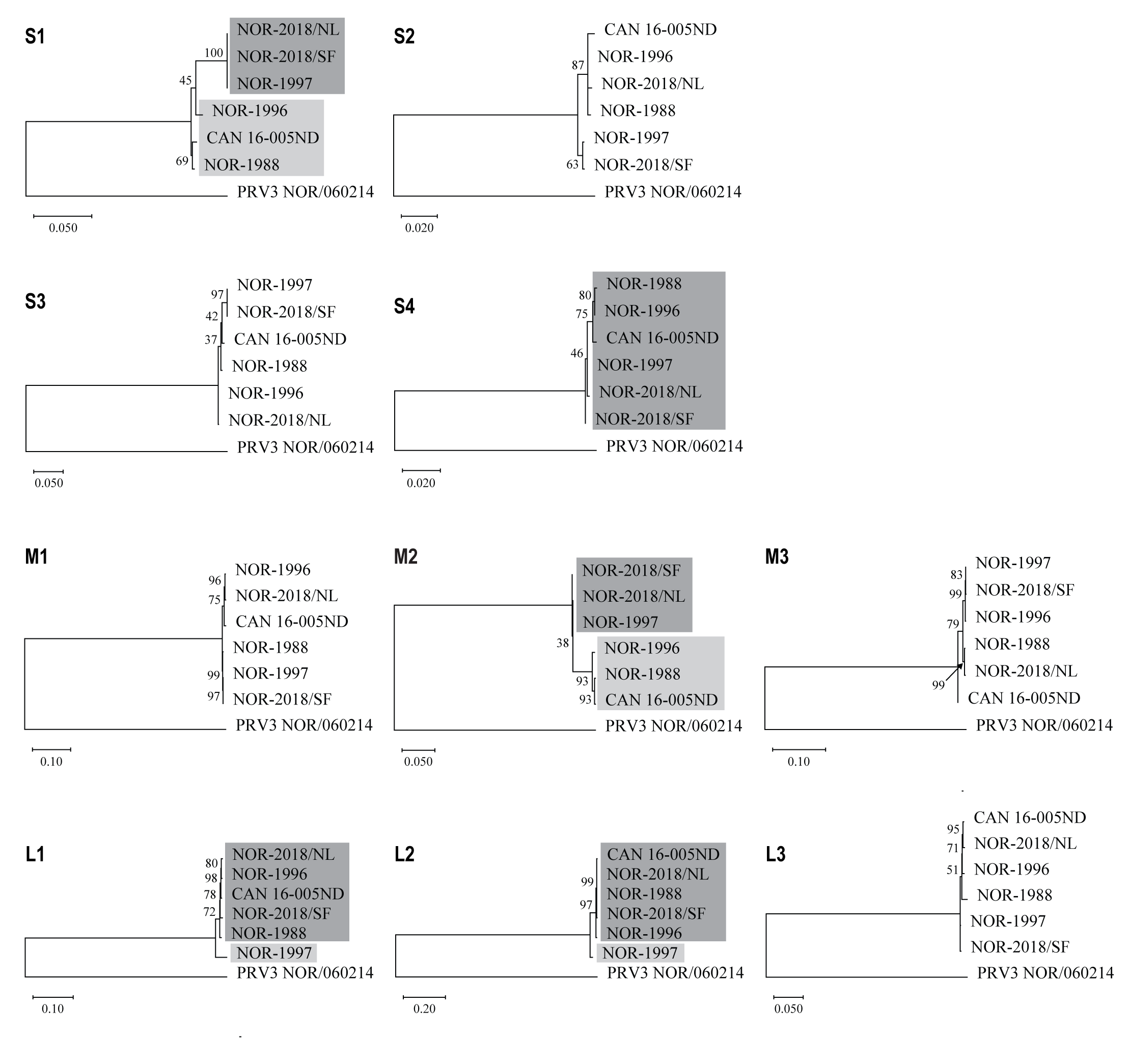


**Figure S11.** Phylogenetic trees constructed from full-length sequences from all ten PRV gene segments using maximum likelihood (ML), for all six isolates studied (NOR-2018/NL, NOR-2018/SF, NOR-1997, NOR-1996, NOR-1988 and CAN 16-005ND). Gene segments potentially linked to virulence are colored grey; dark grey to mark variants associated with higher virulence, light grey for lower virulence. Bootstrap values were calculated from 1000 replicates, and values above 70 can be considered significant. PRV3 strain NOR/060214 (MG253807-MG253816) was selected as outgroup.

**Table S1. Statistical comparison of PRV RNA in plasma.** Statistical analysis comparing mean ranks of Cq-values of RNA in plasma for NOR-2018/SF and NOR-2018/NL, to that of NOR-1997, NOR-1996, NOR-1988 and CAN 16-005ND at 3 and 4 wpc using Mann-Whitney test. Results shown as p-values and marked with asterisk as follows: * p ≤ 0.05, ** p ≤ 0.01).

|  | | NOR-1997 | NOR-1996 | NOR-1988 | CAN 16-005ND |
| --- | --- | --- | --- | --- | --- |
| NOR-2018/SF | 3 wpc | 0.101 | *****  0.024 | *****  0.022 | 0.579 |
|  | 4 wpc | *****  0.041 | 0.670 | 0.159 | 0.516 |
| NOR-2018/NL | 3 wpc | 0.301 | *****  0.029 | ******  0.004 | 0.579 |
|  | 4 wpc | *****  0.041 | 0.796 | 0.591 | 0.986 |

**Table S2. Differences in amino acid sequence between the six isolates.** Amino acids differing from the consensus sequence, or if three isolates differ in sequence at a site (i.e. σ3, p13 and µ1), are shown in red color.

| **Protein (segment)** | **NOR-2018/NL** | **NOR-2018/SF** | **NOR-1997** | **NOR-1996** | **NOR-1988** | **CAN 16-005ND** |
| --- | --- | --- | --- | --- | --- | --- |
| **λ3 (L1)** | A_30_ | A_30_ | T_30_ | A_30_ | A_30_ | A_30_ |
|  | V_63_ | V_63_ | V_63_ | V_63_ | A_63_ | V_63_ |
|  | I_179_ | I_179_ | V_179_ | I_179_ | I_179_ | I_179_ |
|  | V_184_ | V_184_ | A_184_ | V_184_ | V_184_ | V_184_ |
|  | T_200_ | T_200_ | S_200_ | T_200_ | T_200_ | T_200_ |
|  | E_343_ | E_343_ | G_343_ | E_343_ | E_343_ | E_343_ |
|  | D_347_ | D_347_ | E_347_ | D_347_ | D_347_ | D_347_ |
|  | N_372_ | N_372_ | D_372_ | N_372_ | N_372_ | N_372_ |
|  | V_490_ | V_490_ | I_490_ | V_490_ | V_490_ | V_490_ |
|  | N_758_ | N_758_ | S_758_ | N_758_ | N_758_ | N_758_ |
|  | D_937_ | D_937_ | A_937_ | D_937_ | D_937_ | D_937_ |
|  | V_962_ | V_962_ | I_962_ | V_962_ | V_962_ | V_962_ |
|  | N_996_ | N_996_ | S_996_ | N_996_ | N_996_ | N_996_ |
|  | V_1266_ | V_1266_ | I_1266_ | V_1266_ | V_1266_ | V_1266_ |
| **λ2 (L2)** | A_109_ | A_109_ | S_109_ | A_109_ | A_109_ | A_109_ |
|  | D_154_ | D_154_ | N_154_ | D_154_ | D_154_ | D_154_ |
|  | R_412_ | R_412_ | K_412_ | R_412_ | R_412_ | R_412_ |
|  | L_434_ | L_434_ | F_434_ | L_434_ | L_434_ | L_434_ |
|  | A_447_ | A_447_ | T_447_ | A_447_ | A_447_ | A_447_ |
|  | T_932_ | T_932_ | A_932_ | T_932_ | T_932_ | T_932_ |
|  | T_1043_ | T_1043_ | S_1043_ | N_1043_ | T_1043_ | T_1043_ |
|  | V_1055_ | V_1055_ | I_1055_ | V_1055_ | V_1055_ | V_1055_ |
|  | D_1071_ | D_1071_ | E_1071_ | D_1071_ | D_1071_ | D_1071_ |
|  | I_1075_ | I_1075_ | I_1075_ | I_1075_ | I_1075_ | V_1075_ |
|  | A_1128_ | A_1128_ | A_1128_ | A_1128_ | A_1128_ | T_1128_ |
|  | P_1141_ | P_1141_ | L_1141_ | P_1141_ | P_1141_ | P_1141_ |
|  | G_1279_ | G_1279_ | G_1279_ | G_1279_ | G_1279_ | S_1279_ |
| **λ1 (L3)** | A_594_ | A_594_ | A_594_ | A_594_ | S_594_ | A_594_ |
| **µ2 (M1)** | K_113_ | R_113_ | R_113_ | R_113_ | R_113_ | R_113_ |
|  | T_626_ | K_626_ | K_626_ | T_626_ | K_626_ | K_626_ |
| **µ1 (M2)** | T_184_ | T_184_ | T_184_ | S_184_ | S_184_ | S_184_ |
|  | S_262_ | S_262_ | S_262_ | A_262_ | A_262_ | A_262_ |
|  | D_370_ | D_370_ | D_370_ | N_370_ | N_370_ | N_370_ |
|  | V_389_ | V_389_ | V_389_ | V_389_ | A_389_ | V_389_ |

| **µNS (M3)** | L_94_ | M_94_ | M_94_ | M_94_ | L_94_ | M_94_ |
| --- | --- | --- | --- | --- | --- | --- |
|  | R_387_ | R_387_ | R_387_ | R_387_ | R_387_ | H_387_ |
|  | V_451_ | I_451_ | I_451_ | I_451_ | V_451_ | I_451_ |
|  | P_563_ | P_563_ | P_563_ | P_563_ | P_563_ | S_563_ |
|  | H_565_ | H_565_ | H_565_ | H_565_ | H_565_ | R_565_ |
| **σ3 (S1)** | P_39_ | P_39_ | P_39_ | P_39_ | S_39_ | P_39_ |
|  | V_69_ | V_69_ | V_69_ | T_69_ | T_69_ | T_69_ |
|  | D_78_ | D_78_ | D_78_ | E_78_ | E_78_ | E_78_ |
|  | T_85_ | T_85_ | T_85_ | A_85_ | A_85_ | A_85_ |
|  | N_117_ | N_117_ | N_117_ | T_117_ | T_117_ | T_117_ |
|  | V_137_ | V_137_ | V_137_ | I_137_ | I_137_ | I_137_ |
|  | T_156_ | T_156_ | T_156_ | A_156_ | A_156_ | A_156_ |
|  | A_157_ | A_157_ | A_157_ | S_157_ | S_157_ | S_157_ |
|  | E_174_ | E_174_ | E_174_ | K_174_ | K_174_ | K_174_ |
|  | S_180_ | S_180_ | S_180_ | S_180_ | S_180_ | L_180_ |
|  | A_206_ | A_206_ | A_206_ | V_206_ | V_206_ | V_206_ |
|  | V_218_ | V_218_ | V_218_ | I_218_ | I_218_ | I_218_ |
|  | V_230_ | V_230_ | V_230_ | V_230_ | V_230_ | A_230_ |
| **p13 (S1)** | A_16_ | A_16_ | A_16_ | V_16_ | V_16_ | V_16_ |
|  | P_23_ | P_23_ | P_23_ | P_23_ | P_23_ | H_23_ |
|  | M_39_ | M_39_ | M_39_ | T_39_ | T_39_ | T_39_ |
|  | T_50_ | T_50_ | T_50_ | M_50_ | M_50_ | M_50_ |
|  | I_52_ | I_52_ | I_52_ | K_52_ | K_52_ | K_52_ |
|  | V_74_ | V_74_ | V_74_ | A_74_ | A_74_ | A_74_ |
|  | Q_76_ | Q_76_ | Q_76_ | R_76_ | R_76_ | R_76_ |
|  | R_81_ | R_81_ | R_81_ | Q_81_ | Q_81_ | Q_81_ |
|  | M_91_ | M_91_ | M_91_ | L_91_ | L_91_ | L_91_ |
|  | N_93_ | N_93_ | N_93_ | S_93_ | N_93_ | N_93_ |
|  | A_117_ | A_117_ | A_117_ | G_117_ | A_117_ | A_117_ |
| **σ2 (S2)** | V_63_ | V_63_ | V_63_ | V_63_ | V_63_ | I_63_ |
|  | V_393_ | V_393_ | V_393_ | V_393_ | V_393_ | A_393_ |
| **σNS (S3)** | - | - | - | - | - | - |
| **σ1 (S4)** | V_107_ | A_107_ | V_107_ | V_107_ | V_107_ | V_107_ |
|  | N_252_ | D_252_ | D_252_ | D_252_ | D_252_ | D_252_ |

**Table S3.** Viral load in blood cells during PRV-1 propagation. The load of viral RNA and viral σ1-protein in blood cells monitored by RT-qPCR (Cq value) and flow cytometry (MFI) respectively during *in-vivo* propagation of the six PRV-1 isolates NOR-2018/SF, NOR-2018/NL, NOR1997, NOR-1996, NOR-1988, CAN 16-005ND. The sample selected for virus purification from each virus isolate is marked in grey.

|  |  |  | **Load of viral RNA** | | | **Load of viral σprotein** |
| --- | --- | --- | --- | --- | --- | --- |
|  |  |  | RT-qPCR | | | Flow cytometry |
| Group | Time | Fish | Cq (1) | Cq (2) | Cq (mean) | MFI (σ1) |
| **NOR-2018/SF** | 2wpc | F1 | 35.0 | 35.0 | 35.0 | 0.44 |
|  | 2wpc | F2 | 35.0 | 35.0 | 35.0 | 0.43 |
|  | 2wpc | F3 | 35.0 | 35.0 | 35.0 | 0.4 |
|  | 3wpc | F1 | 35.0 | 35.0 | 35.0 | 0.34 |
|  | 3wpc | F2 | 35.0 | 35.0 | 35.0 | 0.33 |
|  | 3wpc | F3 | 35.0 | 35.0 | 35.0 | 0.34 |
|  | 3wpc | F4 | 35.0 | 35.0 | 35.0 | 0.4 |
|  | 4wpc | F1 | 30.5 | 30.4 | 30.4 | 0.42 |
|  | 4wpc | F2 | 25.7 | 25.7 | 25.7 | 0.47 |
|  | 4wpc | F3 | 35.0 | 35.0 | 35.0 | 0.46 |
|  | 4wpc | F4 | 34.8 | 33.9 | 34.4 | 0.43 |
|  | 5wpc | F1 | 15.5 | 15.2 | 15.3 | 3.63 |
|  | 5wpc | F2 | 28.5 | 28.0 | 28.2 | 0.48 |
|  | 5wpc | F3 | 29.7 | 29.6 | 29.6 | 0.47 |
|  | 5wpc | F4 | 18.1 | 17.7 | 17.9 | 1.88 |
|  | 5wpc | F5 | 30.1 | 30.2 | 30.1 | 0.49 |
| **NOR-2018/NL** | 2wpc | F1 | 31.9 | 32.4 | 32.1 | 0.41 |
|  | 2wpc | F2 | 35.0 | 35.0 | 35.0 | 0.47 |
|  | 2wpc | F3 | 33.8 | 33.8 | 33.8 | 0.43 |
|  | 3wpc | F1 | 27.7 | 27.6 | 27.6 | 0.36 |
|  | 3wpc | F2 | 26.8 | 27.1 | 26.9 | 0.41 |
|  | 3wpc | F3 | 21.8 | 22.2 | 22.0 | 0.38 |
|  | 3wpc | F4 | 17.6 | 17.5 | 17.5 | 0.88 |
|  | 4wpc | F1 | 26.9 | 26.9 | 26.9 | 0.45 |
|  | 4wpc | F2 | 30.4 | 30.0 | 30.2 | 0.45 |
|  | 4wpc | F3 | 21.0 | 20.8 | 20.9 | 0.63 |
|  | 4wpc | F4 | 15.7 | 15.8 | 15.7 | 4.57 |
|  | 5wpc | F1 | 15.9 | 14.9 | 15.4 | 3.16 |
|  | 5wpc | F2 | 16.7 | 16.6 | 16.7 | 2.2 |
|  | 5wpc | F3 | 16.4 | 16.2 | 16.3 | 1.87 |
|  | 5wpc | F4 | 26.4 | 27.0 | 26.7 | 0.52 |
|  | 5wpc | F5 | 15.6 | 15.1 | 15.3 | 3.37 |

| **NOR-1997** | 2wpc | F1 | 22.9 | 22.5 | 22.7 | 0,53 |
| --- | --- | --- | --- | --- | --- | --- |
|  | 2wpc | F2 | 26.1 | 26.0 | 26.0 | 0,45 |
|  | 2wpc | F3 | 23.9 | 23.8 | 23.9 | 0,52 |
|  | 3wpc | F1 | 19.4 | 19.5 | 19.4 | 0,56 |
|  | 3wpc | F2 | 14.8 | 14.6 | 14.7 | 2,63 |
|  | 3wpc | F3 | 14.1 | 14.5 | 14.3 | 2,74 |
|  | 3wpc | F4 | 17.4 | 17.1 | 17.2 | 1,05 |
|  | 4wpc | F1 | 17.7 | 17.5 | 17.6 | 4,26 |
|  | 4wpc | F2 | 15.6 | 15.2 | 15.4 | 7,31 |
|  | 4wpc | F3 | 15.5 | 16.0 | 15.8 | 5,31 |
|  | 4wpc | F4 | 16.0 | 15.9 | 15.9 | 5,37 |
|  | 4wpc | F5 | 16.6 | 16.8 | 16.7 | 5,14 |
|  | 4wpc | F6 | 16.5 | 16.2 | 16.4 | 4,15 |
|  | 4wpc | F7 | 17.6 | 17.4 | 17.5 | 3,24 |
|  | 4wpc | F8 | 17.8 | 17.8 | 17.8 | 3,27 |
|  | 4wpc | F9 | 16.1 | 16.2 | 16.1 | 4,57 |
| **NOR-1996** | 2wpc | F1 | 33.0 | 32.9 | 32.9 | 0.44 |
|  | 2wpc | F2 | 34.7 | 35.0 | 34.8 | 0.48 |
|  | 2wpc | F3 | 33.9 | 34.4 | 34.2 | 0.4 |
|  | 3wpc | F1 | 27.1 | 27.3 | 27.2 | 0.36 |
|  | 3wpc | F2 | 27.9 | 28.1 | 28.0 | 0.35 |
|  | 3wpc | F3 | 24.4 | 24.8 | 24.6 | 0.37 |
|  | 3wpc | F4 | 26.9 | 27.1 | 27.0 | 0.37 |
|  | 4wpc | F1 | 27.2 | 27.1 | 27.2 | 0.47 |
|  | 4wpc | F2 | 29.3 | 30.2 | 29.7 | 0.45 |
|  | 4wpc | F3 | 20.7 | 20.6 | 20.7 | 1.93 |
|  | 4wpc | F4 | 15.7 | 15.6 | 15.7 | 7.66 |
|  | 5wpc | F1 | 15.3 | 15.4 | 15.3 | 6.86 |
|  | 5wpc | F2 | 17.0 | 16.5 | 16.7 | 8.29 |
|  | 5wpc | F3 | 15.0 | 15.3 | 15.1 | 5.92 |
|  | 5wpc | F4 | 15.7 | 15.6 | 15.6 | 5.37 |
|  | 5wpc | F5 | 15.7 | 15.3 | 15.5 | 6.12 |

| **NOR-1988** | 2wpc | F1 | 35.0 | 34.8 | 34.9 | 0.49 |
| --- | --- | --- | --- | --- | --- | --- |
|  | 2wpc | F2 | 34.8 | 34.1 | 34.4 | 0.47 |
|  | 2wpc | F3 | 34.4 | 34.1 | 34.2 | 0.48 |
|  | 3wpc | F1 | 27.6 | 27.9 | 27.8 | 0.38 |
|  | 3wpc | F2 | 31.1 | 31.4 | 31.3 | 0.34 |
|  | 3wpc | F3 | 33.7 | 33.2 | 33.4 | 0.32 |
|  | 3wpc | F4 | 27.0 | 26.5 | 26.7 | 0.38 |
|  | 4wpc | F1 | 18.4 | 18.6 | 18.5 | 7.28 |
|  | 4wpc | F2 | 24.5 | 24.4 | 24.4 | 1.09 |
|  | 4wpc | F3 | 18.6 | 18.8 | 18.7 | 7.6 |
|  | 4wpc | F4 | 21.1 | 20.1 | 20.6 | 4.59 |
|  | 5wpc | F1 | 17.9 | 18.0 | 17.9 | 7.33 |
|  | 5wpc | F2 | 18.2 | 17.7 | 18.0 | 7.25 |
|  | 5wpc | F3 | 19.1 | 19.5 | 19.3 | 3.96 |
|  | 5wpc | F4 | 27.3 | 27.3 | 27.3 | 0.51 |
|  | 5wpc | F5 | 19.0 | 18.8 | 18.9 | 6.31 |
| **CAN 16-005ND** | 2wpc | F1 | 28.6 | 28.9 | 28.7 | 0.55 |
|  | 2wpc | F2 | 24.9 | 24.5 | 24.7 | 0.75 |
|  | 2wpc | F3 | 28.1 | 27.5 | 27.8 | 0.48 |
|  | 3wpc | F1 | 20.0 | 19.9 | 20.0 | 2.26 |
|  | 3wpc | F2 | 20.3 | 20.7 | 20.5 | 1.88 |
|  | 3wpc | F3 | 19.7 | 20.0 | 19.9 | 2.49 |
|  | 3wpc | F4 | 20.3 | 20.9 | 20.6 | 1.82 |
|  | 4wpc | F1 | 20.0 | 21.7 | 20.9 | 3.63 |
|  | 4wpc | F2 | 21.3 | 22.3 | 21.8 | 3.81 |
|  | 4wpc | F3 | 20.9 | 20.9 | 20.9 | 4.18 |
|  | 4wpc | F4 | 22.2 | 22.1 | 22.2 | 3.69 |
|  | 4wpc | F5 | 19.9 | 19.3 | 19.6 | 6.51 |
|  | 4wpc | F6 | 20.7 | 20.6 | 20.7 | 2.86 |
|  | 4wpc | F7 | 20.7 | 21.3 | 21.0 | 3.14 |
|  | 4wpc | F8 | 20.7 | 20.7 | 20.7 | 4.63 |
|  | 4wpc | F9 | 22.7 | 24.0 | 23.3 | 2.21 |

**Table S4**. Total number of mapped reads and average coverage mapping of PRV-1 isolates.

|  | **NOR-2018/SF** | | **NOR-2018/NL** | | **NOR-1997** | | **NOR-1996** | | **NOR-1988** | | **CAN 16-005ND** | |
| --- | --- | --- | --- | --- | --- | --- | --- | --- | --- | --- | --- | --- |
| **Segment** | Mapped Reads | Average coverage | Mapped Reads | Average coverage | Mapped Reads | Average coverage | Mapped Reads | Average coverage | Mapped Reads | Average coverage | Mapped Reads | Average coverage |
| **L1** | 7,493 | 72 | 46,465 | 1,294 | 40,056 | 1,627 | 9,544 | 335 | 131,752 | 1,640 | 102,160 | 4,329 |
| **L2** | 7,971 | 76 | 47,889 | 1,381 | 41,983 | 1,744 | 10,422 | 372 | 150,921 | 2,029 | 93,686 | 3,910 |
| **L3** | 7,271 | 73 | 48,772 | 1,537 | 44,973 | 1,763 | 8,836 | 303 | 116,686 | 1,421 | 93,832 | 3,952 |
| **M1** | 3,630 | 64 | 23,902 | 1,258 | 24,012 | 1,533 | 4,990 | 292 | 60,646 | 1,731 | 43,505 | 2,861 |
| **M2** | 3,063 | 65 | 27,377 | 1,810 | 27,780 | 2,113 | 4,476 | 319 | 46,000 | 1,785 | 43,717 | 3,447 |
| **M3** | 3,530 | 63 | 21,493 | 1,112 | 21,300 | 1,371 | 6,813 | 437 | 74,069 | 2,916 | 33,190 | 2,179 |
| **S1** | 2,099 | 118 | 16,001 | 2,247 | 16,907 | 2,634 | 6,418 | 1,058 | 41,943 | 5,492 | 23,865 | 3,763 |
| **S2** | 2,216 | 77 | 17,385 | 1,930 | 17,686 | 2,155 | 3,685 | 440 | 36,401 | 2,602 | 28,283 | 3,508 |
| **S3** | 1,412 | 58 | 12,259 | 1,492 | 9,330 | 1,346 | 3,804 | 539 | 28,563 | 2,529 | 19,893 | 3,002 |
| **S4** | 1,509 | 82 | 13,038 | 1,857 | 13,173 | 2,135 | 4,886 | 804 | 28,316 | 3,086 | 17,621 | 2,817 |

**Table S5.** Primers for immune gene analyses

| **Gene** | **Sequence** | **Accession** |
| --- | --- | --- |
| Elongation factor  (EF)1 α | FP: 5’- TGCCCCTCCAGGATGTCTAC -3’  RP: 5’- TCACCAGGCATAGCCGATTC -3’ | XM_014141923 |
| Interferon  (IFN) 1ab | FP: 5’- ACTGAAACGCTACTTCAAGAAGTTGA -3’  RP: 5’- GCAGATGACGTTTTGTCTCTTTCCT -3’ | AY216595  AY216594 |
| Myxovirius resistance (MX)1 | FP: 5’- GGTGATAGGGGACCAGAGT -3’  RP: 5’- CTCCTCACGGTCTTGGTAGC -3’ | BT043721.1 |
| Interferon-stimulated gene (ISG) 15 | FP: 5’- ATATCTACTGAACATATATCTATCATGGAACTC -3’  RP: 5’- CCTCTGCTTTGTTGTGGCCACTT -3’ | AY795563 |
